# Array-Based CTD Thermal Sensing for Low-Flow Downhole Applications with Dual-Mode Compensation

**DOI:** 10.3390/s26196133

**Published:** 2026-09-27

**Authors:** Zihan Lin, Yong Wei, Yifei Zhang, Ruyi Gan, Mei Wang, Qiang Chen

**Affiliations:** 1 School of Electronic Information and Electrical Engineering, Yangtze University, Jingzhou 434023, China; 2024720709@yangtzeu.edu.cn (Z.L.); 625085@yangtzeu.edu.cn (Y.Z.); ganruyi@yangtzeu.edu.cn (R.G.); meiwang@yangtzeu.edu.cn (M.W.); 2 China Petroleum Logging Co., Ltd., Xi’an 710077, China; qzxq520@163.com

**Keywords:** downhole logging, constant-temperature-difference sensing, thermal flow sensing, sensor array, temperature compensation, PSO–GA–PID control

## Abstract

Accurate downhole temperature sensing and low-flow characterization remain challenging in horizontal and highly deviated wells because of nonuniform cross-sectional flow, temperature drift, and probe-to-probe response differences. This study develops an array-based dual-mode constant-temperature-difference (CTD) sensing system for low-flow downhole applications. The system integrates one upstream temperature-sensing probe and six downstream heated sensing probes with pulse width modulation (PWM)-based CTD regulation, particle swarm optimization–genetic algorithm–proportional–integral–derivative (PSO–GA–PID) parameter tuning, probe-specific polynomial calibration, and depth-matched baseline compensation. The resulting framework is intended to improve the heating stability and consistency of the distributed thermal responses. Simulations using representative CTD thermal dynamics, together with repeated stochastic optimization, show that PSO refinement improves the repeatability of PID parameter tuning and reduces transient deviation. Experimental tests further show that PSO–GA–PID achieves a mean overshoot of 4.34% and a mean integral of time-weighted absolute error (ITAE) of 807.08, both lower than those of GA–PID. After polynomial calibration, the absolute temperature errors of all probes remain within ±0.6 °C over 10–60 °C and ±0.3 °C over 60–150 °C. Under single-phase water conditions, dual-mode baseline compensation reduces the maximum duty-cycle difference among the six sensing probes from 6.1–7.6 to 2.6–4.0 percentage points. The compensated heating duty cycle increases monotonically with flow rate and exhibits higher sensitivity over 0–30 m^3^/d than over 30–60 m^3^/d, providing a measurable basis for subsequent flow-rate calibration. Additional water-cut experiments show that the duty-cycle–flow relationship depends on fluid composition, indicating the need for composition-specific calibration under oil–water two-phase conditions. These results demonstrate the feasibility of the integrated array-based CTD sensing and compensation framework for stable multipoint thermal response characterization under controlled low-flow conditions.

## 1. Introduction

The exploitation of low-permeability oilfields has increased steadily, with such reservoirs now accounting for 49% of all producing oilfields [1]. In these reservoirs, downhole production-profile monitoring remains technically challenging because these wells commonly exhibit low fluid velocities, high water cuts, and complex flow behavior in irregular wellbores [2,3]. Downhole temperature and flow-rate measurements are essential for evaluating well productivity, identifying producing zones, and supporting intelligent zonal water injection [4,5]. Nevertheless, accurate temperature and flow-rate measurement under low-flow-rate conditions remains difficult, particularly when cross-sectional spatial resolution is required in horizontal or highly deviated wells.

Conventional turbine, ultrasonic, and electromagnetic flowmeters have been widely used in general industrial flow measurement. However, their applicability to low-flow-rate measurements under harsh downhole conditions may be constrained by mechanical response thresholds, signal attenuation, installation requirements, and long-term reliability concerns [6,7,8]. Thermal flowmeters are therefore a promising alternative for low-flow measurement because of their simple structure, lack of moving parts, and high sensitivity to low fluid velocities [9]. Their response, however, depends strongly on the thermal environment, temperature measurement accuracy, and stability of the heating-control loop.

Previous studies have addressed these issues from different perspectives. Zou et al. proposed a strong-tracking, fast-convergence adaptive robust Kalman filter for thermal gas flowmeters based on microelectromechanical systems (MEMS) and reduced the dynamic response time by 83.3% [10]. Yan et al. developed a modified Sage–Husa adaptive robust strong-tracking Kalman filter, reducing both the root-mean-square error and mean absolute error by more than 75% [11]. For liquid-flow applications, Feng et al. developed a low-power thermal flowmeter for long-term downhole monitoring over 1–15 m^3^/d [12]. Jia et al. implemented a thermal liquid flowmeter based on a digital signal processor (DSP), using constant-temperature-difference (CTD) and thermal-diffusion principles with incremental proportional–integral–derivative (PID) temperature control over 0–5 m^3^/d [13], while Dai et al. employed conventional PID regulation and pulse width modulation (PWM)-driven digital heating to improve the dynamic response of a thermal liquid flowmeter [14]. These studies demonstrate the feasibility of thermal sensing for low-flow measurement and the importance of stable heating regulation.

Temperature-induced output variation has also been addressed through circuit-, sensor-, and model-level compensation. Wang et al. developed a dual-winding multipoint CTD thermal gas sensor with linear temperature compensation [15]. Gao et al. established a semi-empirical temperature-effect model incorporating the thermophysical properties of the fluid and sensor structure [16]. Qi et al. compensated for temperature-dependent heating-power variation using a digitally adjusted CTD circuit [17], whereas Sosna et al. employed a dual-Wheatstone-bridge circuit to reduce the influence of medium-temperature fluctuations [18]. Wu et al. combined cubic spline interpolation and polynomial regression to compensate for temperature drift over a wide temperature range [19]. These approaches mainly improve individual sensing elements, readout circuits, or single-sensor thermal models under varying temperature conditions.

For an array-based downhole thermal sensor, these problems become coupled. Multiple heated probes are required to obtain distributed flow-sensitive responses across the wellbore cross-section, while probe-to-probe differences may introduce different temperature baselines and heating responses. At the same time, flow-dependent convection alters the dynamic behavior of the CTD heating loop. Therefore, the performance of such a system depends not only on the thermal-sensing principle itself, but also on the coordinated design of the probe array, heating regulation, temperature calibration, and baseline compensation.

To address these coupled requirements, this study develops an array-based dual-mode CTD sensing system for low-flow downhole applications. The sensing section combines one upstream temperature-sensing probe with six downstream heated probes to obtain circumferentially distributed flow-sensitive responses. The probe arrangement is designed for the intended 5.5-inch casing environment by balancing cross-sectional coverage and thermal interaction. A genetic algorithm (GA) is first used to tune the PID parameters, followed by particle swarm optimization (PSO) refinement to improve CTD temperature regulation under varying thermal conditions. Probe-specific polynomial calibration and depth-matched dual-mode baseline compensation are then used to reduce temperature-related differences among the sensing probes.

The experimental evaluation follows the measurement chain of the developed system. Probe-level temperature accuracy is first characterized, followed by single-channel CTD tests to evaluate heating-control performance. The six-probe array is then tested under controlled flow conditions to assess probe-to-probe consistency and the duty-cycle response to changes in flow rate. Additional water-cut experiments are used to examine the influence of fluid composition on the thermal response, while the principal uncertainty sources in the measurement chain are also evaluated. The main contributions of this work are summarized as follows:

(1) An array-based dual-mode CTD sensing architecture is implemented for low-flow downhole applications by coordinating one upstream temperature reference with six downstream heated sensing probes, providing distributed flow-sensitive responses within the targeted 5.5-inch casing environment.
(2) A coordinated temperature-control and compensation workflow is established for the sensing array. The two-stage PSO–GA–PID tuning procedure, consisting of GA-based global search followed by PSO refinement, improves CTD heating regulation, while probe-specific polynomial calibration and depth-matched baseline compensation reduce temperature-related differences among the sensing probes.
(3) The developed system is experimentally characterized from temperature acquisition to array-level thermal response. The experiments quantify temperature-measurement accuracy, controller performance, probe-to-probe duty-cycle consistency, flow-dependent sensitivity, water-cut dependence, and the principal uncertainty sources of the measurement chain.

Section 2 describes the measurement principles, array structure, and dual-mode measurement strategy. Section 3 presents the PID parameter-tuning procedure used for CTD temperature regulation, and Section 4 evaluates its performance through simulation. Section 5 introduces the polynomial calibration and dual-mode baseline compensation method. Section 6 presents the experimental results, and Section 7 concludes the article.

## 2. Measurement Principles and Methods

### 2.1. Dual-Mode Array Measurement Scheme

Horizontal and highly deviated wells commonly develop nonuniform velocity distributions because gravity-driven phase segregation and asymmetric flow redistribute fluid around the wellbore circumference. A single-point measurement may therefore represent only the local condition at one position. Array-turbine measurements in inclined gas–water wells have also shown distinct responses among circumferentially distributed probes under nonuniform flow conditions [20], illustrating the value of spatially distributed observations for resolving local flow variations. The sensing section consequently uses a circumferential array to resolve spatial variations within the targeted 5.5-inch casing, while the dual-mode scheme allows the same probes to acquire the temperature field before CTD flow sensing.

As shown in Figure 1, the sensing section consists of one upstream temperature-sensing probe and six downstream velocity-sensing probes. The upstream probe, denoted as P7, is located on the upstream centerline and is not heated. It supplies a common ambient-temperature reference to all six CTD loops, placing the heated channels on the same temperature basis. Because P7 encounters the fluid before the downstream heated plane, it tracks the incoming local temperature, while the axial separation is intended to reduce direct thermal interference from the micro-heaters. The six velocity-sensing probes, denoted as P1–P6, are arranged uniformly on the downstream measurement plane with approximately 60° angular spacing, enabling distributed sensing of circumferentially varying thermal responses.

The instrument therefore uses six velocity probes (P1–P6) distributed around the pipe circumference at approximately 60° intervals and one upstream temperature probe (P7). A larger number of circumferential probes can provide finer angular sampling of local thermal-response variations but increases the requirements for installation space, probe separation, wiring, heating power, and independent control. Within the spatial and system-integration constraints of the current instrument, six heated probes were selected to balance circumferential sampling against implementation complexity. The probe count was not determined through a systematic optimization study.

The array tests reported in Section 6.3 further show that the six-channel arrangement can operate as an integrated measurement system. All six channels were controlled simultaneously over the tested flow-rate range, and polynomial calibration combined with dual-mode baseline compensation limited the maximum inter-probe duty-cycle difference to 2.6–4.0 percentage points. The resulting channel consistency allows the six local thermal responses to be interpreted together during subsequent flow characterization.

The upstream reference probe is positioned approximately 30 cm ahead of the velocity-probe plane. This axial spacing fits the available instrument envelope and is intended to reduce direct thermal interference from the downstream micro-heaters while retaining a local upstream temperature reference. The 30 cm distance is a geometric parameter of the present prototype; its thermal-isolation performance and optimality were not systematically evaluated.

The two operating modes correspond to successive stages of the field measurement. During temperature logging, all micro-heaters remain off, and the seven probes continuously record the temperature distribution while the instrument moves through the target interval. Real-time depth from the surface logging system identifies the prescribed flow-measurement stations. At each selected depth, axial tool motion is stopped, and the latest unheated measurements establish the local temperature baseline before CTD flow sensing. The operating sequence is illustrated in Figure 2.

The surface control system then electronically enables flow mode. The six micro-heaters and their independent CTD loops are activated without changing the fixed probe arrangement. Each heated probe maintains the prescribed temperature difference relative to P7, and its steady-state PWM duty cycle is recorded as a local flow-sensitive output. After the measurement is completed, a surface command switches off the heaters, the instrument returns to temperature mode, and motion resumes toward the next selected depth. This procedure is repeated at the measurement stations specified by the logging program.

### 2.2. Temperature Reference Measurement

The temperature-reference measurement provides the ambient-temperature input for CTD control and the baseline information for probe-level compensation. PT1000 platinum resistance thermometers are embedded in all seven sensing probes. The operating temperature range of the sensing section is −30 °C to 175 °C [21]. The PT1000 resistance–temperature relationship is modeled by the Callendar–Van Dusen equation [22]:

(1) $$R_{T}=R_{0}[1+AT+BT^{2}+C\left(T-100\right)T^{3}]$$

where *R_T_* is the PT1000 resistance at temperature *T*; *R*_0_ is the nominal resistance at 0 °C (*R*_0_ = 1000 Ω); and *A*, *B*, and *C* are the Callendar–Van Dusen coefficients. In this study, *A* = 3.9083 × 10^−3^ °C^−1^, *B* = −5.775 × 10^−7^ °C^−2^, and *C* = −4.183 × 10^−12^ °C^−4^. The *C* term is included only for the subzero portion of the operating range. A constant-current-source (CCS) readout is used for the seven PT1000 measurement channels. As shown in Figure 3, a stable current is applied to each PT1000 sensor, and the voltage across the sensor is sampled. The excitation current is fixed at 1 mA. The corresponding electrical dissipation in channel j is *P_self_*_,*j*_ = $I_{S}^{2}$*R_T_*_,*j*_. The Callendar–Van Dusen resistance range therefore gives 0.88–1.67 mW over the full −30 to 175 °C operating interval, or 1.08–1.57 mW over the 20–150 °C interval used in the self-heating test. This readout dissipation is distinct from the actively controlled micro-heater power used during flow measurement.

For the *j*-th temperature-reading channel, the resistance is obtained as:

(2) $$R_{tj}=\frac{U_{sj}}{I_{s}},j=1,2,\cdots ,7$$

where *U_sj_* is the measured voltage of the *j*-th channel. The corresponding temperature *T_j_* is then calculated from (1).

The temperature rise of the packaged probes was evaluated experimentally because the effective dissipation coefficient depends on sensor mounting and the heat-transfer boundary condition. All seven probes were held in a quiescent calibration bath at 20, 60, 100, and 150 °C and measured at excitation currents of 0.25, 0.50, 0.75, 1.00, and 1.25 mA. For each probe and bath temperature, the indicated-temperature offset relative to the reference thermometer was fitted as Δ*T_j_* = *a_j_* + *b_j_*$I_{s}^{2}$; the intercept represents the zero-current offset, and the current-dependent term gives the self-heating rise. The coefficient of determination (R^2^) ranged from 0.944 to 0.997. At the 1 mA operating current, the fitted temperature rise was 0.024–0.047 °C across the seven probes and four bath temperatures. The corresponding mean rises were 0.029, 0.033, 0.038, and 0.043 °C at 20, 60, 100, and 150 °C, respectively.

These measurements quantify the self-heating contribution associated with the adopted 1 mA excitation; they do not establish that its effect on CTD temperature-difference measurement is negligible. The repeatable contribution under calibration conditions is included in the probe-specific calibration of the complete measurement chain, while the unheated measurements obtained immediately before flow sensing correct the initial probe-to-reference offset at each depth. Because the upstream reference and heated probes may experience different local convective cooling, differential self-heating need not cancel completely; its possible effect on the regulated PWM duty cycle is retained as a condition-dependent systematic influence in the uncertainty discussion in Section 6.6.

The reference temperature measured by the upstream probe at depth *z* is denoted as *T_n_*(*z*). During flow measurement, *T_n_*(*z*) defines the CTD control target for the six heated velocity-sensing probes and serves as the local temperature reference in the CTD sensing model. Probe-specific calibration and temperature-drift compensation procedures are presented in Section 5.

### 2.3. CTD-Based Local Flow-Sensing Principle

The developed instrument employs the constant temperature difference (CTD) principle to obtain flow-sensitive thermal responses [23,24,25,26]. During CTD operation, each downstream velocity-sensing probe is independently heated and controlled to maintain a prescribed temperature difference relative to the upstream temperature-sensing probe.

Figure 4 illustrates one CTD sensing unit. The upstream temperature-sensing probe provides the ambient reference temperature *T_n_*. The *i*-th downstream velocity-sensing probe integrates a heater and a PT1000 sensor, and its measured temperature is denoted as *T_mi_*. The temperature difference of each heated probe is defined below.

The velocity-sensing probes retain the integrated construction developed and validated in previous work. Each probe comprises a 61 mm long, 6 mm diameter beryllium-copper shell with a wall thickness of 0.5 mm. A 20 mm PT1000 element and a 20 mm 0Cr21Al6 alloy heater are mounted within the shell, and the surrounding space is filled with hexagonal boron nitride (BN) to provide electrical insulation and thermal coupling to the shell. Detailed internal assembly and component arrangement are reported in Ref. [27] and documented in Chinese Patent CN114705262B [28]. The array instrument uses this probe without changes to its geometry or materials. These established features determine the effective thermal capacitance, conduction path, and convective boundary conditions represented by the heat-transfer relations below and the lumped thermal model in Section 4, while the array implementation provides multichannel CTD regulation, dual-mode baseline compensation, and coordinated flow sensing.

(3) $$\Delta T_{i}=T_{mi}-T_{n}=\Delta T_{set},i=1,2,\cdots ,6$$

where Δ*T_set_* is the preset temperature difference maintained by the CTD controller.

At steady state, the heating power required to maintain Δ*T_set_* is mainly determined by the convective heat transfer from the heated sensing probe to the surrounding fluid. The corresponding heat-transfer relationship can be expressed as [29]:

(4) $$P_{i}=h_{i}S_{i}\Delta T_{set},i=1,2,\cdots ,6$$

where *P_i_* is the heating power of the *i*-th velocity-sensing probe, *h_i_* is the convective heat-transfer coefficient, and *S_i_* is the effective heat-transfer area. Approximating the heated section of the probe as a cylinder with effective length *l_i_* and diameter *d_i_*, *S_i_* = *πl_i_d_i_*. By introducing the Nusselt number *Nu_i_* = *h_i_d_i_*/*λ_f_*, Equation (4) becomes:

(5) $$P_{i}=\pi l_{i}\lambda_{f}Nu_{i}\Delta T_{set},i=1,2,\cdots ,6$$

For forced convection around a cylindrical probe, the Kramers correlation is used to describe the heat-transfer trend [30]:

(6) $$Nu_{i}=0.42Pr^{0.2}+0.57Pr^{0.33}Re_{i}^{0.5},i=1,2,\cdots ,6$$

where *P_r_* = *μc_p_*/*λ_f_* and *Re_i_* = *ρv_i_d_i_*/*μ*. Here, *λ_f_*, *μ*, *c_p_*, and *ρ* denote the fluid thermal conductivity, dynamic viscosity, specific heat capacity, and density, respectively; and *v_i_* represents the local velocity at the *i*-th velocity-sensing probe. Substituting Equation (6) into Equation (5), the relationship between heating power and local velocity can be expressed as:

(7) $$P_{i}=\pi l_{i}\lambda_{f}\Delta T_{set}\left[0.42Pr^{0.2}+0.57Pr^{0.33}{\left(\frac{\rho v_{i}d_{i}}{\mu }\right)}^{0.5}\right],i=1,2,\cdots ,6$$

During a stationary measurement at a selected depth, the fluid properties are treated as locally stable over the measurement interval after the temperature baseline has been obtained. The terms associated with probe geometry and fluid properties can therefore be grouped into two probe-dependent coefficients, giving:

(8) $$P_{i}=\left(k_{1i}+k_{2i}v_{i}^{0.5}\right)\Delta T_{set},i=1,2,\cdots ,6$$

where *k*_1*i*_ and *k*_2*i*_ represent the velocity-independent and velocity-dependent contributions to the effective heat-transfer response, respectively.

Equation (8) provides a physically interpretable basis for relating the required CTD heating power to local flow velocity. In the actual sensing section, however, the thermal field can deviate from ideal cylindrical cross-flow because of wall confinement, probe-support structures, packaging geometry, installation tolerances, and local thermal-fluid conditions. Therefore, *k*_1*i*_ and *k*_2*i*_ are treated in practical implementation as effective probe-dependent parameters to be identified or corrected through flow calibration for the target fluid and operating range, rather than as universal constants determined solely from the ideal correlation.

Accordingly, the analytical relationship in Equation (8) is used to preserve the expected physical dependence between convection, heating demand, and flow velocity, while practical deviations of the implemented sensor are handled through subsequent calibration. Significant changes in wellbore geometry, fluid composition, temperature range, or flow regime may therefore require corresponding recalibration.

In the developed CTD system, the heating power is regulated through the PWM duty cycle. As described in Section 2.4, the PWM duty cycle provides a measurable representation of the heating demand required to maintain Δ*T_set_*. Combining the CTD heat-transfer relationship with the PWM-based heating-power characterization gives the empirical calibration form:

(9) $$v_{i}=A_{1i}\theta_{i}^{2}-A_{2i}\theta_{i}+A_{3i},i=1,2,\cdots ,6$$

where *θ_i_* is the PWM duty cycle, and *A*_1*i*_, *A*_2*i*_, and *A*_3*i*_ are probe-dependent calibration coefficients.

Equation (9) defines a potential calibration framework for converting duty-cycle response into local velocity estimation. In practical application, these coefficients should be determined from calibration data for the target fluid and operating conditions. In the present study, the experimentally measured PWM duty cycles are therefore used primarily to characterize the flow-sensitive response of the sensing array and to provide measurable inputs for subsequent quantitative flow-rate calibration.

### 2.4. PWM-Based Heating-Power Characterization and Multichannel Output

In the developed CTD system, PWM signals are used to regulate the heating power of the micro-heaters [31,32]. Let *θ_i_* denote the PWM duty cycle of the *i*-th velocity-sensing probe expressed as a percentage. When the heater voltage during the on state is *U_t_*, the root mean square (RMS)-equivalent voltage applied to the heater is:

(10) $$U_{ei}=U_{t}\sqrt{\frac{\theta_{i}}{100}}$$

where *θ_i_*/100 represents the duty ratio.

For a resistive heater with resistance Ri, the equivalent heating power is:

(11) $$P_{i}=\frac{U_{ei}^{2}}{R_{i}}=\frac{\left(\theta_{i}/100\right)U_{t}^{2}}{R_{i}},i=1,2,\cdots ,6$$

Equation (11) indicates that the PWM duty cycle provides a measurable representation of the heater power required to maintain the CTD temperature difference. Since the six velocity-sensing probes are independently controlled, each probe generates an individual duty-cycle response corresponding to its local flow condition.

In practical implementation, differences in probe fabrication and installation may cause variations in the initial temperature response among sensing probes. The temperature-related baseline deviations are compensated through polynomial temperature calibration and dual-mode baseline compensation, as described in Section 5. After compensation, the six duty-cycle outputs provide distributed flow-sensitive information for subsequent flow-rate calibration.

## 3. Temperature Control Algorithm Design

CTD measurement requires each heated velocity-sensing probe to maintain a prescribed temperature difference relative to the upstream reference probe. Because variations in flow rate modify convective heat transfer and consequently change the equivalent thermal dynamics of the heating loop, fixed PID gains may not provide consistent transient performance over the full operating range. Therefore, a two-stage PSO-enhanced GA strategy is used to optimize the PID gains for CTD temperature regulation.

### 3.1. Incremental PID Control

The continuous PID control law is expressed as [33,34]:

(12) $$u\left(t\right)=K_{p}e\left(t\right)+K_{i}\int_{0}^{t}e(\tau )\;d\tau +K_{d}\frac{de\left(t\right)}{dt}$$

where *u*(*t*) is the control output; *e*(*t*) is the temperature-difference tracking error; and *K_p_*, *K_i_*, and *K_d_* are the proportional, integral, and derivative gains, respectively. The corresponding discrete incremental form is:

(13) $$\Delta u(k)=K_{p}\left[e(k)-e(k-1)\right]+K_{i}T_{s}e(k)+\frac{K_{d}}{T_{s}}\left[e(k)-2e(k-1)+e(k-2)\right]$$

where *T_s_* is the sampling period. The PWM duty-cycle command is updated as:

(14) $$u(k)=sat\left[u(k-1)+\Delta u(k)\right]$$

where sat[·] constrains the controller output to the allowable PWM range of 0–100%. Since fixed empirical gains may provide inconsistent performance under varying thermal conditions, the PID parameters are optimized using the following two-stage strategy.

### 3.2. Composite-Objective-Based PSO–GA–PID Optimization

The parameter vector is defined as:

(15) $$x=\left[K_{p},K_{i},K_{d}\right]$$

For each candidate vector *x*, the closed-loop temperature response is evaluated using the equivalent CTD thermal model. To account for tracking accuracy, transient peak suppression, and PWM-output smoothness, a composite objective function is adopted:

(16) $$J(x)=\bar{\mathrm{ITAE}}+w_{os,m}\bar{M_{p}^{2}}+w_{os,\max}M_{p,\max}^{2}+w_{du}\bar{D_{u}}$$

where $\bar{ITAE}$ is the mean ITAE over the considered flow-rate conditions, $\bar{M_{p}}$ is the overshoot percentage, *M_p_*_,max_ is the maximum overshoot among all evaluated conditions, and *D_u_* is the PWM duty-cycle variation. The weighting coefficients are set to *w_os_*_,*m*_ = 25, *w_os_*_,max_ = 120, and *w_du_* = 0.0015, based on preliminary convergence tests and magnitude balancing among the objective terms [35]. The maximum-overshoot term was assigned a relatively larger weight to limit worst-case transient peaks, while the PWM-variation term was assigned a smaller weight to avoid excessive smoothing of the control response.

For each flow-rate condition, ITAE is calculated in discrete form as:

(17) $$\mathrm{ITAE}=T_{s}\sum_{k=0}^{N-1}t_{k}\left|e\left(k\right)\right|$$

where *N* is the number of samples and *t_k_* = *kT_s_*. The PWM duty-cycle variation is calculated as:

(18) $$D_{u}=T_{s}\sum_{k=1}^{N-1}{\left[u\left(k\right)-u\left(k-1\right)\right]}^{2}$$

A smaller value of *J*(*x*) indicates better aggregate control performance. Based on preliminary CTD-loop simulations and commonly used ranges in evolutionary PID tuning, the PID search ranges are defined as:

(19) $$K_{p}\in \left[0,250\right],K_{i}\in \left[0,15\right],K_{d}\in \left[0,20\right]$$

The proposed optimization strategy combines the global search capability of GA with the local refinement capability of PSO. GA is first used to explore the PID parameter space within the bounds in (19). Each individual represents one candidate PID gain vector. The population size and maximum number of generations are set to 50 and 100, respectively, and the crossover and mutation probabilities are set to 0.6 and 0.1, respectively. Candidate individuals are evaluated using (16), and selection, crossover, mutation, and elitist preservation are performed iteratively to retain promising PID gain vectors.

After the GA search, PSO is used to refine the candidate solutions. The best GA solution is retained as an elite particle, and the remaining particles are initialized using selected GA individuals and their perturbed variants. The swarm size and maximum number of PSO iterations are set to 30 and 100, respectively, and the acceleration coefficients are set to *c*_1_ = *c*_2_ = 2.0. The inertia weight decreases linearly from 0.9 to 0.6 during the PSO iterations. These algorithmic settings were selected based on preliminary convergence tests and commonly used parameter ranges in evolutionary PID tuning [36].

For the *i*-th particle at iteration *ξ*, the velocity and position are updated as follows:

(20) $$\left\{\begin{array}{l} v_{i}^{\left(\xi +1\right)}=wv_{i}^{\xi }+c_{1}r_{1}\left(p_{i}-x_{i}^{\xi }\right)+c_{2}r_{2}\left(g-x_{i}^{\xi }\right)\\ x_{i}^{\left(\xi +1\right)}=x_{i}^{\xi }+v_{i}^{\left(\xi +1\right)} \end{array}\right.$$

where *x_i_^ξ^* and *v_i_^ξ^* are the position and velocity of particle *i* at iteration *ξ*, respectively; *p_i_* is the personal best position of particle *i*; *g* is the global best position of the swarm; *c*_1_ and *c*_2_ are acceleration coefficients; and *r*_1_ and *r*_2_ are independent random variables uniformly distributed in [0, 1]. The inertia weight is linearly decreased as:

(21) $$w_{\xi }=w_{\max}-\frac{\left(w_{\max}-w_{\min}\right)\xi }{K_{\max}}$$

where *w_ξ_* is the inertia weight at iteration *ξ*; *w*_max_ and *w*_min_ are the initial and final inertia weights, respectively; and *K*_max_ is the maximum number of PSO iterations. In this study, *w*_max_ and *w*_min_ are set to 0.9 and 0.6, respectively.

After each position update, particles exceeding the prescribed PID bounds are clipped to the nearest feasible boundary. Each particle’s objective value is then recalculated using (16), and the personal and global best positions are updated accordingly. Upon reaching the maximum number of iterations, the final global-best particle is selected as the optimized PID parameter vector for the incremental PID controller.

In this two-stage framework, GA provides diverse candidate PID gains through global exploration, whereas PSO further refines the search around promising regions. GA–PID and PSO–GA–PID use the same PID bounds, model-evaluation procedure, and composite objective function. Therefore, the performance difference between GA–PID and PSO–GA–PID can be mainly attributed to the additional PSO refinement. The controller architecture and complete optimization procedure are shown in Figure 5 and Figure 6, respectively.

## 4. Simulation Verification of the Temperature Control Algorithm

### 4.1. Simulation Model and Parameter Settings

The closed-loop CTD simulations were implemented in MATLAB version 9.11 (R2021b) using a script-based discrete-time model. At each prescribed volumetric flow-rate condition *Q_q_*, the reference signal *r*(*t*) is compared with the model output *y_q_*(*t*) to form the tracking error *e_q_*(*t*) = *r*(*t*) − *y_q_*(*t*). The incremental PID controller described in Section 3.1 converts this error into the actuator command *u_q_*(*t*), which is applied to the flow-dependent plant *G_q_*(*s*). The resulting temperature-difference output is returned through the feedback path.

To evaluate the relative temperature-control performance of the three controllers under flow-dependent thermal dynamics, a second-order oscillatory model with pure time delay was adopted as an equivalent model of the CTD heating loop [37]. The model represents the dominant effects of thermal inertia, response delay, and flow-dependent convection and is used as a common simulation basis for controller comparison rather than as a direct experimental identification of the complete instrument. For the *q*-th flow-rate condition, the equivalent thermal model is expressed as:

(22) $$G_{q}\left(s\right)=\frac{K_{q}\omega_{n,q}^{2}e^{-L_{q}s}}{s^{2}+2\zeta_{q}\omega_{n,q}s+\omega_{n,q}^{2}}$$

Under zero initial conditions, Equation (22) has the input–output relationship:

(23) $$G_{q}(s)=\frac{Y_{q}(s)}{U_{q}(s)}$$

The equivalent delayed time-domain equation is:

(24) $$y_{q}^{\prime \prime }(t)+2\zeta_{q}\omega_{n,q}y_{q}^{\prime }(t)+\omega_{n,q}^{2}y_{q}(t)=K_{q}\omega_{n,q}^{2}u_{q}(t-L_{q})$$

In Equations (22)–(24), *Q_q_* is the prescribed volumetric flow rate for condition *q* (m^3^/d). It is an external operating variable, rather than a controller output, and selects the parameter set in Table 1. The equivalent thermal gain *K_q_* has units of °C/%, *ω_n_*_,*q*_ is the natural frequency in rad/s, *ζ_q_* is the dimensionless damping ratio, and *L_q_* is the delay in seconds. The plant output *y_q_*(*t*) = Δ*T_q_*(*t*) = *T_m_*_,*q*_(*t*) − *T_n_*_,*q*_(*t*) is the temperature difference between the heated probe and the upstream reference probe. The reference *r*(*t*) is the prescribed temperature-difference signal, and the actuator input *u_q_*(*t*) is the PWM duty-cycle command expressed as a percentage from 0 to 100, not the flow rate or heating power itself.

With increasing flow rate, stronger convection reduces the equivalent thermal gain, while the natural frequency and damping ratio increase moderately to represent the corresponding variation in thermal response. The parameters listed in Table 1 are therefore used as representative simulation settings for controller comparison rather than as experimentally identified model parameters.

All controller comparisons used prescribed flow rates of 0, 5, 10, 15, 20, 25, 30, 40, 50, and 60 m^3^/d; a sampling period of 0.2 s; a duration of 600 s; and a 0.5 °C step reference applied at *t* = 0. The model was initialized in an unheated equilibrium state, with *y_q_*(0) = 0 °C, *y*^′^*_q_*(0) = 0 °C/s, and *u_q_*(*t*) = 0% for *t* < 0. The zero initial temperature difference represents the absence of active-heating temperature rise, rather than an absolute sensor temperature of 0 °C. The model therefore describes the baseline-corrected CTD response and does not reproduce the calibration and baseline-compensation procedures outlined in Section 5.

For numerical integration, the continuous state vector was defined by *x*_1_ = *y_q_* and *x*_2_ = *y*^′^*_q_*. The state equation corresponding to Equation (24) was discretized by zero-order hold at each flow condition, with the discrete state matrices calculated from the matrix exponential. Because *L_q_* = 4.0 s and *T_s_* = 0.2 s, the pure delay was represented by *d* = *L_q_*/*T_s_* = 20 input samples, and the pre-simulation input history was filled with zeros. At each sample, the incremental PID equations in (13) and (14) updated *u_q_*[*k*] and constrained it to 0–100%; the plant state was then advanced using the delayed command *u_q_*[*k* − *d*]. No additional anti-windup correction was applied beyond this output saturation. The sampling sequence, initial state, delay history, and plant parameters were identical for PID, GA-PID, and PSO-GA-PID comparisons.

Three controllers were evaluated under identical conditions: conventional PID, GA–PID, and PSO–GA–PID. The baseline PID gains were set to *K_p_* = 3, *K_i_* = 4, and *K_d_* = 0, which were selected through preliminary simulations to ensure stable tracking without persistent PWM saturation. The derivative gain was set to zero because derivative action did not further improve the baseline response in the preliminary tests [38]. The optimized gain sets for GA–PID and PSO–GA–PID were obtained using the procedures described in Section 3 and were applied to all simulated flow-rate conditions without retuning.

Because GA and PSO are stochastic optimization algorithms, both optimization procedures were independently repeated 20 times using different random initial populations. For each run, the optimized *K_p_*, *K_i_*, and *K_d_*, together with the final objective-function value, were recorded. Their mean values and standard deviations were used to evaluate the repeatability of the optimization results.

### 4.2. Simulation Results and Discussion

The statistical convergence characteristics of GA–PID and PSO–GA–PID over 20 independent optimization runs are shown in Figure 7. The curves represent the mean best-so-far objective value, with the corresponding run-to-run variation expressed by ±1 standard deviation. Both methods converge to stable solutions. However, PSO–GA–PID exhibits noticeably smaller variation among independent runs, indicating that the additional PSO refinement improves the repeatability of the stochastic parameter search.

The repeated-run statistics are summarized in Table 2. For GA–PID, the optimized gains are *K_p_* = 2.7567 ± 0.2957, *K_i_* = 1.9495 ± 0.0036, and *K_d_* = 13.3481 ± 1.7198, with a final objective value of 1329.2275 ± 0.1184. For PSO–GA–PID, the corresponding values are *K_p_* = 2.9795 ± 0.0399, *K_i_* = 1.9525 ± 0.0003, and *K_d_* = 13.4726 ± 0.6473, with a final objective value of 1329.10748 ± 0.00167. Although the mean final objective values of the two methods are close, PSO–GA–PID yields substantially smaller standard deviations in both the optimized gains and the final objective value, demonstrating improved optimization consistency.

For the subsequent controller comparison, the run whose final objective value was closest to the mean value of the 20 independent runs was selected for each optimization method. The resulting representative gains were *K_p_* = 2.5650, *K_i_* = 1.9478, and *K_d_* = 12.4813 for GA–PID, and *K_p_* = 2.9926, *K_i_* = 1.9531, and *K_d_* = 12.8764 for PSO–GA–PID. These representative parameter sets were used for the simulations shown in Figure 8 and Figure 9 and for the performance indices reported in Table 3.

Figure 8 shows the simulated temperature-difference step responses of the three controllers at representative flow rates of 5, 20, and 30 m^3^/d. Both optimized controllers exhibit substantially improved damping and stabilization compared with the conventional PID controller. The responses of GA–PID and PSO–GA–PID are similar during the initial rise, whereas PSO–GA–PID exhibits smaller transient deviation and faster stabilization.

The quantitative results in Table 3 further confirm this tendency. Compared with GA–PID, PSO–GA–PID increases the mean rise time from 28.78 s to 33.67 s, but reduces the mean settling time from 49.44 s to 39.67 s. More importantly, the mean overshoot decreases from 3.993% to 2.341%, and the maximum overshoot decreases from 4.826% to 2.894%. The mean ITAE is also reduced from 539.15 to 486.63, corresponding to a reduction of approximately 9.74%. These results indicate that, under the adopted representative CTD thermal models, the PSO refinement primarily improves transient damping and accumulated-error suppression rather than response speed.

Figure 9 compares the ITAE values of GA–PID and PSO–GA–PID over all ten simulated flow-rate conditions, including 0, 5, 10, 15, 20, 25, 30, 40, 50, and 60 m^3^/d. The lower overall ITAE obtained by PSO–GA–PID is consistent with the mean results in Table 3, indicating improved cumulative tracking-error suppression over the considered flow range.

Overall, the simulation results demonstrate the relative optimization characteristics of the three control strategies under the adopted representative CTD thermal models. The PSO refinement improves the transient suppression capability and accumulated tracking-error performance compared with GA–PID within the evaluated simulation conditions. These results indicate that the PSO–GA–PID strategy provides improved controller robustness under the adopted representative CTD thermal dynamics. The practical temperature-control performance of the optimized controller is further evaluated using the experimental CTD sensing system in Section 6.

## 5. Temperature Calibration and Baseline Compensation for Array-Based Dual-Mode Measurement

Although the proposed controller improves CTD heating stability, probe-dependent temperature drift may still affect array-based CTD sensing. Since the steady-state PWM duty cycle serves as the measurable input for subsequent flow-rate calibration, temperature-reading errors and baseline offsets among sensing probes can be transferred to the duty-cycle output. Therefore, this section introduces a compensation method combining probe-specific polynomial calibration and dual-mode baseline correction. The former corrects nonlinear temperature-reading errors, while the latter compensates for the baseline offset between each velocity-sensing probe and the upstream reference probe before heater activation.

### 5.1. Effect of Temperature-Measurement Drift on CTD Response

Temperature-measurement drift, calibration residuals, and probe-to-probe baseline differences can shift the temperature difference used to initialize CTD regulation [39]. Here, Δ*T_e_* denotes the unheated temperature-difference bias between the *i*-th velocity-sensing probe and the upstream reference probe before micro-heater activation. It is defined from temperature-mode measurements and is distinct from the closed-loop tracking error *e*(*t*) of the PID controller. According to the CTD heating-power model in (8), the influence of an uncompensated bias can be expressed as:

(25) $$P_{i}=\left(k_{1i}+k_{2i}v_{i}^{0.5}\right)\left(\Delta T_{set}+\Delta T_{e,i}\right),i=1,2,\cdots ,6$$

Equation (25) maps the pre-CTD temperature-difference bias to the steady heating demand. If the bias is not corrected before flow mode, it shifts the effective temperature difference after the loop settles and changes the required heating power and PWM duty cycle. The equation therefore describes the effect of a measurement bias on the regulated steady state rather than the transient tracking error of the closed loop. Section 5.3 applies the depth-matched baseline offset obtained in temperature mode to correct this probe-dependent bias before heater activation.

### 5.2. Polynomial Calibration of Sensor Probes

A temperature calibration experiment was conducted to establish a probe-specific correction model for each sensing probe. As shown in Figure 10, the sensing section was immersed in No. 46 industrial white oil (Mobil, Exxon Mobil Corporation, Spring, TX, USA) while retaining the relative probe geometry used by the instrument. The six velocity-sensing probes were positioned in one horizontal measurement plane, and the temperature-sensing probe was approximately 30 cm below this plane. The independent reference sensor connected to the temperature controller (Fujian Shunchang Hongrun Precision Instrument Co., Ltd., Shunchang, Nanping, Fujian, China) was placed near the bottom of the oil tank, close to the temperature-sensing probe, and its reading was used as the calibration reference.

The controller set the oil bath to successive target temperatures from 10 to 150 °C. After each target was reached, the setpoint was maintained for approximately 2 min before data acquisition. No mechanical stirring or forced circulation was used. Preliminary tests showed an observable transient difference between the velocity-probe plane and the lower temperature-sensing location immediately after a setpoint change, whereas no appreciable difference was observed after the 2 min stabilization period. Measurements were therefore recorded only after the sensing region had thermally stabilized; spatial uniformity outside this region was not assumed.

The temperatures of the seven sensing probes and the independent reference sensor were recorded synchronously at 1 s intervals. The complete calibration experiment lasted approximately 130 min, yielding approximately 7800 synchronized time samples. Each time sample comprised one simultaneous record of all eight temperature channels rather than a sum of individual channel readings. All channels used the same 1 mA excitation, so the fitted probe-specific models characterize the complete measurement chain, including repeatable PT1000 readout self-heating under the calibration-bath condition.

Each synchronized record contained the raw temperature reading of each sensing probe and the corresponding time-matched reference temperature. For each probe, the raw probe temperature was used as the regression input and the reference temperature as the target. The probe-specific cubic-polynomial coefficients were estimated by least-squares fitting. The fitted temperatures were then compared with the corresponding reference temperatures to calculate the coefficient of determination (R^2^) and root-mean-square error (RMSE) reported in Table 4 and Table 5.

To balance calibration accuracy and computational complexity, a cubic polynomial regression model was adopted:

(26) $$T=\alpha_{0}+\alpha_{1}x+\alpha_{2}x^{2}+\alpha_{3}x^{3}$$

where *T* denotes the reference temperature; *x* denotes the temperature measured by a sensing probe; and *α*_0_, *α*_1_, *α*_2_, and *α*_3_ are the polynomial coeffici00ents. The fitted equations and evaluation metrics of all probes are listed in Table 4 and Table 5. The probe-specific polynomial coefficients were determined exclusively from the calibration dataset and were fixed before the subsequent temperature-validation and repeatability measurements.

The fitted equations in Table 4 and the evaluation metrics in Table 5 indicate that the cubic polynomial model fits the tested temperature range well. Nevertheless, residual temperature differences may still exist among probes under the same temperature field. These residual differences can affect CTD heating control and reduce consistency among the sensing probes during array-based CTD sensing. Therefore, a dual-mode baseline compensation mechanism is introduced to correct probe-dependent baseline deviations after polynomial calibration.

### 5.3. Dual-Mode Baseline Compensation Strategy

The dual-mode compensation strategy uses the temperature-mode data acquired immediately before flow measurement to correct probe-dependent baseline offsets. When the instrument reaches the selected depth *z*, the calibrated temperature of each velocity-sensing probe is compared with that of the upstream reference probe:

(27) $$\Delta T_{ci}(z)=T_{i}(z)-T_{7}(z),i=1,2,\cdots ,6$$

where Δ*T_ci_* is the baseline offset of the *i*-th velocity-sensing probe, *T_i_*(*z*) is the calibrated temperature of the *i*-th velocity-sensing probe, and *T*_7_(*z*) is the calibrated temperature of the upstream reference probe. This offset is applied before heater activation to correct the initial baseline mismatch of each velocity-sensing probe. The compensated probes are then heated and independently controlled in flow mode to maintain the prescribed temperature difference. The overall dual-mode baseline compensation procedure is illustrated in Figure 11.

## 6. Experimental Results and Analysis

Figure 12 shows the prototype of the array-based dual-mode temperature–flow logging instrument, including the sensing section, probe arrangement, and control circuit boards. The experimental validation follows the measurement sequence of the developed system. Temperature-measurement accuracy and repeatability are first evaluated to verify the reliability of the sensing probes. The dynamic performance of the CTD heating loop is then examined using a single-channel test. Subsequently, the six-probe array is tested to evaluate the effect of dual-mode baseline compensation on probe-to-probe consistency. Finally, the compensated duty-cycle responses are characterized under different flow rates and water-cut conditions.

### 6.1. Temperature Calibration, Accuracy, and Repeatability

The validation data were acquired in a separate experimental run and were not used for polynomial fitting or coefficient selection. The fixed calibration equations were applied to the newly acquired probe readings. For each synchronized record, the measurement error was calculated as the calibrated probe temperature minus the corresponding reference temperature. These errors were used to construct Figure 13, where probes 1–6 are the velocity-sensing probes and probe 7 is the upstream temperature-sensing probe.

After polynomial calibration, the absolute temperature errors of all probes remain within ±0.6 °C over 10–60 °C and within ±0.3 °C over 60–150 °C. The relatively larger errors at lower temperatures may be associated with stronger transient thermal nonuniformity during the initial heating stage, whereas the error distributions become more concentrated at higher temperatures. Because the validation retained the packaged sensors, 1 mA excitation, and acquisition electronics, these bounds describe the system-level residual after calibration and already include the repeatable readout self-heating present under the validation conditions.

Repeatability was evaluated with the calibration coefficients from Section 5.2 held fixed. Three independent temperature cycles were performed at 20, 60, 100, and 150 °C, with each bath condition re-established in every cycle and maintained at the target setpoint for approximately 2 min before acquisition. The seven calibrated probe outputs were then recorded for 60 s at 1 s intervals. At each temperature, the 60 readings from each probe were first averaged within each independent cycle. For each probe–temperature combination, the sample standard deviation (*n* − 1 denominator, *n* = 3) and range were then calculated from the three cycle means. Table 6 reports the arithmetic mean of the seven probe-specific standard deviations, the largest probe-specific standard deviation, and the largest probe-specific range at each temperature. The three-cycle records used for Table 6 formed a separate repeatability dataset; they were not used to refit the polynomial coefficients or to construct the pointwise validation-error curves in Figure 13.

Across the four temperatures, the mean repeatability standard deviation among the seven probes was 0.029–0.043 °C. The maximum standard deviation for any probe–temperature combination was 0.061 °C, and the largest run-to-run range was 0.12 °C. These results show that the calibrated readings remain consistent when the bath condition is independently re-established.

Together, the accuracy and repeatability results show that the calibrated probe array provides stable temperature measurements over the tested range and establishes the temperature basis for subsequent baseline compensation and CTD operation.

### 6.2. Experimental Validation of Temperature Control

A single-channel CTD test platform consisting of one temperature-sensing probe and one velocity-sensing probe was constructed to evaluate the closed-loop temperature-control performance. The experimental setup is shown in Figure 14. This configuration isolates one CTD heating loop so that the influence of the controller can be evaluated before the array-level experiments.

The water pump was adjusted to establish flow-rate conditions of 0, 5, 10, 15, 20, 25, 30, 40, 50, and 60 m^3^/d, consistent with the simulation conditions. Under each condition, PID, GA–PID, and PSO–GA–PID were tested using the same prescribed temperature difference of 0.5 °C. The real-time temperature difference was recorded, and representative measured responses at 5, 20, and 30 m^3^/d are shown in Figure 15. The quantitative indices averaged over all tested conditions are summarized in Table 7.

The performance indices were calculated from the experimentally recorded temperature-difference time series for each controller at each prescribed flow rate. The tracking error was defined as the 0.5 °C setpoint minus the measured temperature difference. Rise time was the elapsed time required for the response to first reach 95% of the setpoint, and settling time was the first time after which all subsequent samples remained within ±5% of the setpoint. Overshoot was evaluated relative to the 0.5 °C setpoint, and the discrete integral of time-weighted absolute error was calculated as ITAE = ∑*_k_ t_k_*|*e_k_*|Δ*t* using the actual acquisition interval Δ*t* = 1 s. The same criteria were applied to all controllers. The mean values in Table 7 are arithmetic averages of the respective indices across the ten flow-rate conditions, whereas maximum overshoot is the largest value among those conditions. Figure 15 presents the measured responses at 5, 20, and 30 m^3^/d as representative examples.

Both optimized controllers improve the CTD heating response compared with conventional PID. Compared with GA–PID, PSO–GA–PID reduces the mean overshoot from 7.95% to 4.34%, the maximum overshoot from 8.96% to 5.66%, and the mean ITAE from 1231.84 to 807.08. Its mean settling time is also reduced from 68.19 s to 56.41 s. These results show that the PSO refinement mainly improves transient suppression and accumulated tracking-error performance under practical flow disturbances.

To evaluate the repeatability of the measured CTD control performance, three independent heating runs were conducted at 5, 20, and 30 m^3^/d with GA–PID and PSO–GA–PID. The temperature-difference setpoint and controller gains were held constant, and the initial thermal condition was re-established before each run. Rise time, settling time, overshoot, and ITAE were calculated separately from each recorded response using the definitions given above. Table 8 reports the arithmetic mean and sample standard deviation of each index across the three runs at each flow rate.

Across the six flow-rate–controller combinations, the sample standard deviation was 0.58–3.46 s for rise time, 0.58–1.53 s for settling time, 0.10–0.40 percentage points for overshoot, and 8.26–24.62 for ITAE. At 5, 20, and 30 m^3^/d, PSO–GA–PID retained lower mean settling time, overshoot, and ITAE than GA–PID across the repeated measurements.

### 6.3. Compensation Effect on Consistency Among Sensing Probes

After the single-channel CTD control performance was verified, the six-probe array was tested to evaluate the effect of dual-mode baseline compensation on sensing consistency.

The experiment was conducted using the CNPC oil–water flow test facility shown in Figure 16. The facility provides controllable flow-rate and water-cut conditions in a simulated wellbore.

For the single-phase water experiment, the calibrated instrument was installed in the simulated wellbore at room temperature. The prescribed temperature difference was 0.5 °C, and the water cut was fixed at 100%. The reference flow rate was increased from 0 to 60 m^3^/d in increments of 5 m^3^/d. At each condition, the instrument operated in flow mode for 5 min to reach a steady response, and the stabilized PWM duty-cycle reading of each velocity-sensing probe was recorded at the end of the 5 min measurement period. The same procedure was performed with polynomial calibration only and after applying dual-mode baseline compensation. The results are shown in Figure 17 and Table 9.

For each flow-rate condition, the mean duty cycle was calculated as the arithmetic mean of the six probe outputs, and the maximum inter-probe difference was calculated as their maximum minus minimum, in percentage points. These quantities were calculated separately for the polynomial-calibration-only and dual-mode-compensated conditions and are presented in Table 9. A smaller maximum inter-probe difference indicates better probe-to-probe consistency.

With polynomial calibration only, the duty-cycle difference ranges from 6.1 to 7.6 percentage points. After applying dual-mode baseline compensation, it decreases to 2.6–4.0 percentage points. The reduction is maintained over the tested flow-rate range, indicating that the pre-heating baseline correction effectively reduces temperature-related differences among the sensing probes and improves the consistency of the array duty-cycle response.

The repeatability of the array response was evaluated using three independent measurements at 0, 20, and 30 m^3^/d under single-phase water conditions. Each run included a separate unheated baseline acquisition followed by CTD heating at the 0.5 °C setpoint. Under each compensation condition, the stabilized PWM duty cycles of all six velocity-sensing probes were recorded at the end of the 5 min measurement period. For each run, the array mean duty cycle and maximum inter-probe duty-cycle difference were calculated from the six probe outputs. Their arithmetic means and sample standard deviations across the three runs are summarized in Table 10.

The across-run standard deviation of the array mean duty cycle was 0.10 percentage points under all six tested conditions. With polynomial calibration only, the mean maximum inter-probe difference was 6.17–7.27 percentage points (sample SD = 0.40 percentage points). After baseline compensation, it was 3.17–3.97 percentage points (sample SD = 0.31–0.40 percentage points). The lower inter-probe spread was reproduced in all three measurements at each tested flow rate.

The remaining differences among probes may also be influenced by structural and local flow-field variations, which are not addressed by the temperature-baseline compensation.

### 6.4. Duty-Cycle Response to Flow Rate

The compensated duty-cycle data obtained under single-phase water conditions were further analyzed to characterize the response of the sensing array to flow-rate variation. As shown in Figure 17 and Table 9, the steady-state heating duty cycle increases monotonically with flow rate for all six velocity-sensing probes. This behavior is consistent with the CTD principle because stronger convection removes more heat from the heated probe and therefore requires a higher heating duty cycle to maintain the prescribed temperature difference.

The response exhibits an approximately piecewise linear trend. In the 0–30 m^3^/d range, the fitted duty-cycle sensitivities of the six probes are 0.78–0.82%/(m^3^/d). In the 30–60 m^3^/d range, the sensitivities decrease to 0.31–0.36%/(m^3^/d). The larger slopes at lower flow rates indicate stronger sensitivity of the duty-cycle response in the low-flow region. For each flow-rate interval, duty-cycle sensitivity was obtained as the slope of a separate least-squares linear regression of steady-state duty cycle against reference flow rate for each probe.

The monotonic duty-cycle response provides a suitable measurable input for subsequent flow-rate calibration. In practical use, the calibration relationship should be established using calibration data for the target fluid and operating range, with independent flow points used to assess the resulting inversion accuracy.

### 6.5. Influence of Water Cut on Heating Duty Cycle

The influence of fluid composition was further investigated using water cuts of 100%, 80%, 60%, 40%, 20%, and 0%. For each water-cut condition, the reference flow rate was increased from 0 to 60 m^3^/d in increments of 5 m^3^/d, and the steady-state PWM duty cycles of all six velocity-sensing probes were recorded after polynomial calibration and dual-mode baseline compensation. The results are shown in Figure 18.

At the same flow rate, the heating duty cycle decreases as the water cut decreases. This trend indicates that the CTD response depends on both flow rate and fluid composition because changes in density, viscosity, thermal conductivity, and specific heat capacity alter the convective heat-transfer process.

Accordingly, the duty-cycle–flow relationship is composition dependent. The single-phase water response obtained in Section 6.4 is directly applicable to water-flow characterization, while oil–water mixtures require calibration corresponding to their fluid composition. For applications in which the water cut is known, composition-specific calibration curves can be established. When the water cut is unknown, additional composition information or a coupled flow–composition model is required.

These results show that the array is sensitive to both flow-rate and fluid-composition variations and define the calibration requirements for extending the method from single-phase to oil–water two-phase conditions.

### 6.6. Measurement-Chain Uncertainty Assessment and Error Sources

The reliability of the CTD sensing response depends on the complete measurement chain, including temperature acquisition, excitation-current stability, digital conversion, PWM heating regulation, heater characteristics, and the reference flow condition. The principal quantified or specified uncertainty sources are summarized in Table 11. The analysis is performed at the measurement-chain level because several component-level effects are already incorporated into the experimentally calibrated sensor response.

For a quantity specified by a symmetric tolerance of ±*a*, and in the absence of additional information on its probability distribution, a rectangular distribution is assumed. The corresponding standard uncertainty is therefore:

(28) $$u\left(x\right)=\frac{a}{\sqrt{3}}$$

For digitally quantized quantities with a discrete step Δ*x*, the quantization error is bounded by ±Δ*x*/2. Assuming a uniform distribution within one quantization interval, the corresponding standard uncertainty is:

(29) $$u_{q}\left(x\right)=\frac{\Delta x}{\sqrt{12}}$$

These relations are used below to obtain the component-level standard uncertainties listed in Table 11.

The temperature-measurement chain is based on the PT1000 resistance–temperature relationship described in Section 2.2. Since the resistance is obtained from *R* = *U*/*I*, the standard uncertainty associated with an uncalibrated resistance measurement can, assuming independent voltage and current contributions, be expressed by first-order uncertainty propagation as:

(30) $${\left(\frac{u_{R}}{R}\right)}^{2}={\left(\frac{u_{U}}{U}\right)}^{2}+{\left(\frac{u_{I}}{I}\right)}^{2}$$

The excitation-current stability is specified as 0.10% of the nominal current. Under the rectangular distribution assumption, its relative standard uncertainty is therefore:

(31) $$u_{I,\mathrm{rel}}=\frac{0.10\%}{\sqrt{3}}=0.0577\%$$

The temperature-readout circuit employs a 24-bit analog-to-digital converter (ADC). If *V*_FS_ denotes the effective full-scale input range under the actual acquisition configuration, the ADC quantization step can be written as:

(32) $$q_{ADC}=\frac{V_{FS}}{2^{24}}$$

The corresponding quantization standard uncertainty is:

(33) $$u_{ADC}=\frac{q_{ADC}}{\sqrt{12}}$$

The resulting voltage and current uncertainties propagate to the resistance estimate and subsequently to temperature through the PT1000 resistance–temperature relationship. However, the probe-level polynomial calibration in Section 5.2 is performed using the complete acquisition chain, including the PT1000 element, excitation-current source, ADC, and readout circuitry. Therefore, systematic contributions from the excitation and acquisition circuits are largely incorporated into the experimentally determined calibration coefficients and should not be independently recombined with the observed calibration residuals, as this would result in double counting. The repeatable PT1000 self-heating at the fixed 1 mA excitation is included in the same residual and is therefore not added again as an independent uncertainty term.

The experimental temperature-validation results provide a system-level measure of the residual temperature uncertainty after calibration. The absolute residuals remain within ±0.6 °C over 10–60 °C and ±0.3 °C over 60–150 °C. When these maximum observed residuals are conservatively treated as bounded intervals, the corresponding standard uncertainties are:

(34) $$\left\{\begin{array}{l} u_{T,10-60}=\frac{0.60}{\sqrt{3}}=0.346\;{}^{\circ}C\\ u_{T,60-150}=\frac{0.30}{\sqrt{3}}=0.173\;{}^{\circ}C \end{array}\right.$$

The independent temperature cycles in Section 6.1 provide a separate Type A measure of run-to-run precision, with a maximum repeatability standard deviation of 0.061 °C. This statistic is reported separately from the absolute validation residuals because the two quantities describe different aspects of the same calibrated measurement chain.

The reference temperature sensor has an accuracy of ±0.10 °C, corresponding to:

(35) $$u_{T,\mathrm{ref}}=\frac{0.10}{\sqrt{3}}=0.0577\;{}^{\circ}C$$

Because the experimentally observed calibration residuals are obtained by comparison with this reference measurement, these quantities are reported separately in Table 11 rather than combined into a single propagated temperature uncertainty.

For the heating-control path, the implemented PWM duty-cycle step is 0.10 percentage points. The quantization error is therefore bounded within ±0.05 percentage points, and the corresponding standard uncertainty is:

(36) $$u_{\theta }=\frac{0.10}{\sqrt{12}}=0.0289$$

According to Equation (11), the equivalent heater power can be expressed as:

(37) $$P_{i}=\frac{U_{t}^{2}}{R_{i}}\frac{\theta_{i}}{100}$$

For independent input quantities, the first-order relative uncertainty relationship is:

(38) $${\left(\frac{u_{P_{i}}}{P_{i}}\right)}^{2}={\left(2\frac{u_{U_{t}}}{U_{t}}\right)}^{2}+{\left(\frac{u_{\theta_{i}}}{\theta_{i}}\right)}^{2}+{\left(\frac{u_{R_{i}}}{R_{i}}\right)}^{2}$$

The sensitivity coefficient of the heater voltage term is two because the heating power is proportional to the square of the voltage. This relationship shows directly how the heater-voltage stability, PWM quantization, and resistance measurement affect the equivalent heating-power estimate. In the present study, the available data allow the PWM contribution and the inter-probe heater-resistance variation to be characterized, whereas a separate statistical uncertainty for the heater-voltage measurement was not determined; therefore, a combined heating-power uncertainty is not reported.

The measured heater resistances of the six velocity-sensing probes are 22.64, 22.27, 22.69, 22.34, 21.76, and 21.54 Ω. Their mean value is:

(39) $$\bar{R}=\frac{1}{6}\sum_{i=1}^{6}R_{i}=22.207\Omega$$

The sample standard deviation is:

(40) $$s_{R}=\sqrt{\frac{\sum_{i=1}^{6}{\left(R_{i}-\bar{R}\right)}^{2}}{6-1}}=0.466\Omega$$

The total range is 1.15 Ω, corresponding to approximately 5.2% of the mean heater resistance. This quantity represents an actual inter-probe difference rather than a resistance-measurement uncertainty. Because *R_i_* appears explicitly in the heating-power relationship, the measured resistance of each probe can be used individually during calibration or heating-power characterization instead of treating the six heaters as identical. The current manuscript indeed reports heater resistances of 21.54–22.69 Ω and identifies their influence on the duty-cycle-to-heating-power relationship.

The reference flowmeter used to define the experimental flow conditions has an accuracy of ±0.5% of reading. Assuming a rectangular distribution, the corresponding relative standard uncertainty is:

(41) $$u_{Q,\mathrm{rel}}=\frac{0.5\%}{\sqrt{3}}=0.289\%$$

This uncertainty applies to the imposed reference flow condition and therefore affects the flow-rate coordinate used when interpreting the measured duty-cycle response.

Probe positioning and fluid thermophysical properties represent additional systematic influences that cannot presently be separated into independent numerical uncertainty components. The relative positions of the upstream reference probe and the six heated probes are determined by the mechanical assembly, and deviations in their axial or circumferential positions may modify the local velocity and thermal fields. The probe-positioning uncertainty was therefore not independently quantified and is treated as a structural influence in the present assessment. Similarly, thermal conductivity, viscosity, density, and specific heat capacity affect the convective heat-transfer relationship in Equations (6)–(8). Their combined influence is also reflected by the composition-dependent duty-cycle responses observed in Section 6.5. These effects should therefore be incorporated through condition-specific calibration when the system is applied to different fluids or operating environments.

The multi-current test in Section 2.2 bounds the PT1000 self-heating rise under quiescent calibration-bath conditions at 0.024–0.047 °C for the 1 mA readout. This interval describes the magnitude of the measured effect rather than a separate standard uncertainty. During CTD operation, changes in local convection can alter heat removal differently at the upstream and heated probes, leaving a differential component that is not fully removed by common-mode cancelation or by the preheating baseline. Until dynamic self-heating is separated experimentally from other environmental effects, it is treated as a condition-dependent systematic influence and should be reassessed when the fluid, flow regime, or probe installation changes.

Overall, the above analysis provides a measurement-chain uncertainty assessment rather than a fully propagated flow-rate uncertainty budget. The quantified terms establish the uncertainty basis for temperature acquisition, PWM regulation, heater characterization, and the imposed reference flow condition, while probe geometry and fluid-property variations are retained as systematic influences. A complete propagated uncertainty of an inferred flow rate would additionally require a validated duty-cycle–flow calibration model and the corresponding sensitivity coefficients.

## 7. Conclusions

In this study, an array-based dual-mode CTD sensing system was developed for low-flow downhole applications. The system coordinates one upstream temperature-sensing probe and six downstream heated sensing probes with PWM-based CTD regulation, PSO–GA–PID tuning, probe-specific polynomial calibration, and depth-matched baseline compensation. The emphasis of the work is on the coordinated sensing architecture, temperature-control strategy, and compensation workflow required for stable array operation under low-flow conditions.

The main conclusions are as follows.

(1) The optimized controllers improve the dynamic performance of the CTD heating loop compared with conventional PID. In the experimental tests, PSO–GA–PID reduces the mean overshoot from 7.95% to 4.34%, the maximum overshoot from 8.96% to 5.66%, and the mean ITAE from 1231.84 to 807.08 compared with GA–PID. These results indicate improved transient suppression and accumulated tracking-error performance under the tested flow disturbances. Across three independent runs at each of 5, 20, and 30 m^3^/d, the controller ordering was maintained, with PSO–GA–PID yielding lower mean settling time, overshoot, and ITAE than GA–PID.

(2) Probe-specific polynomial calibration improves the temperature-measurement consistency of the sensing array. After calibration, the absolute temperature errors of all probes remain within ±0.6 °C over 10–60 °C and within ±0.3 °C over 60–150 °C, providing a stable temperature basis for CTD regulation and subsequent baseline compensation. Across three independent temperature cycles, the mean repeatability standard deviation among the seven probes was 0.029–0.043 °C, the maximum individual-probe standard deviation was 0.061 °C, and the maximum run-to-run range was 0.12 °C.

(3) The dual-mode baseline compensation reduces temperature-related differences among the six velocity-sensing probes. Under single-phase water conditions, the maximum duty-cycle difference among the probes decreases from 6.1–7.6 to 2.6–4.0 percentage points after compensation, demonstrating improved array-level response consistency. In the repeatability tests at 0, 20, and 30 m^3^/d, the sample standard deviation of the array mean duty cycle was 0.10 percentage points, while the mean maximum inter-probe difference decreased from 6.17–7.27 percentage points before compensation to 3.17–3.97 percentage points after compensation.

(4) Under single-phase water conditions, the compensated heating duty cycle increases monotonically with the imposed flow rate. The response exhibits higher sensitivity over 0–30 m^3^/d than over 30–60 m^3^/d, indicating stronger flow sensitivity in the lower flow range. These duty-cycle characteristics provide the measurement basis for subsequent flow-rate calibration.

(5) The water-cut experiments show that the duty-cycle–flow relationship depends on fluid composition. Therefore, calibration established under single-phase water conditions cannot be directly transferred to oil–water mixtures with different compositions. Composition-specific calibration is required when the water cut is known, whereas unknown two-phase conditions require additional composition information or a coupled flow–composition model.

(6) The uncertainty analysis identifies the principal quantifiable contributions from temperature acquisition, excitation-current stability, ADC and PWM quantization, heater-resistance variation, and the reference flow condition. These results provide the uncertainty basis for interpreting the measured CTD temperature and duty-cycle responses.

Overall, the experimental results demonstrate that the developed array-based CTD system can provide stable temperature regulation, improved probe-to-probe consistency, and monotonic flow-sensitive duty-cycle responses under controlled laboratory conditions. The present results establish the sensing and calibration basis for subsequent quantitative flow-rate estimation. Future work will focus on independent flow-rate calibration and validation, high-temperature and high-pressure testing, long-term stability, and composition-dependent calibration for oil–water two-phase conditions.

## Figures and Tables

**Figure 1:**
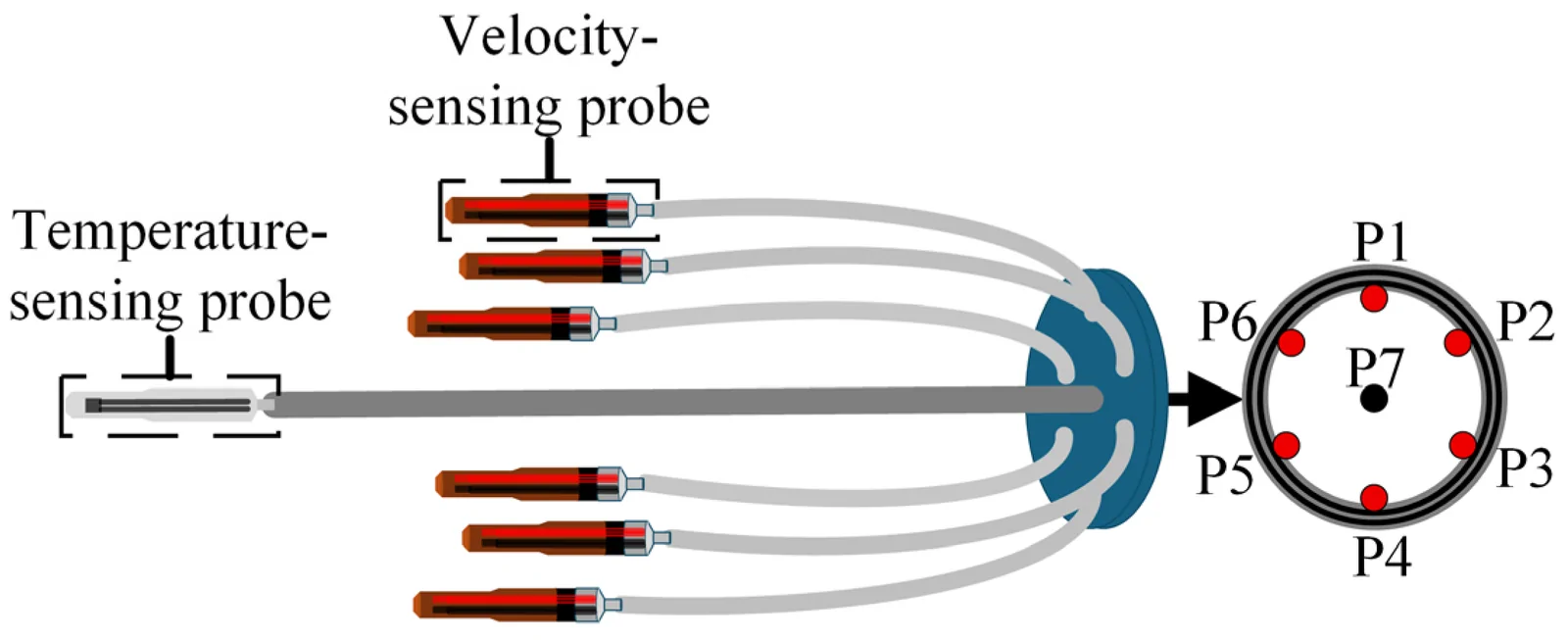
Array-based sensing-section structure.

**Figure 2:**
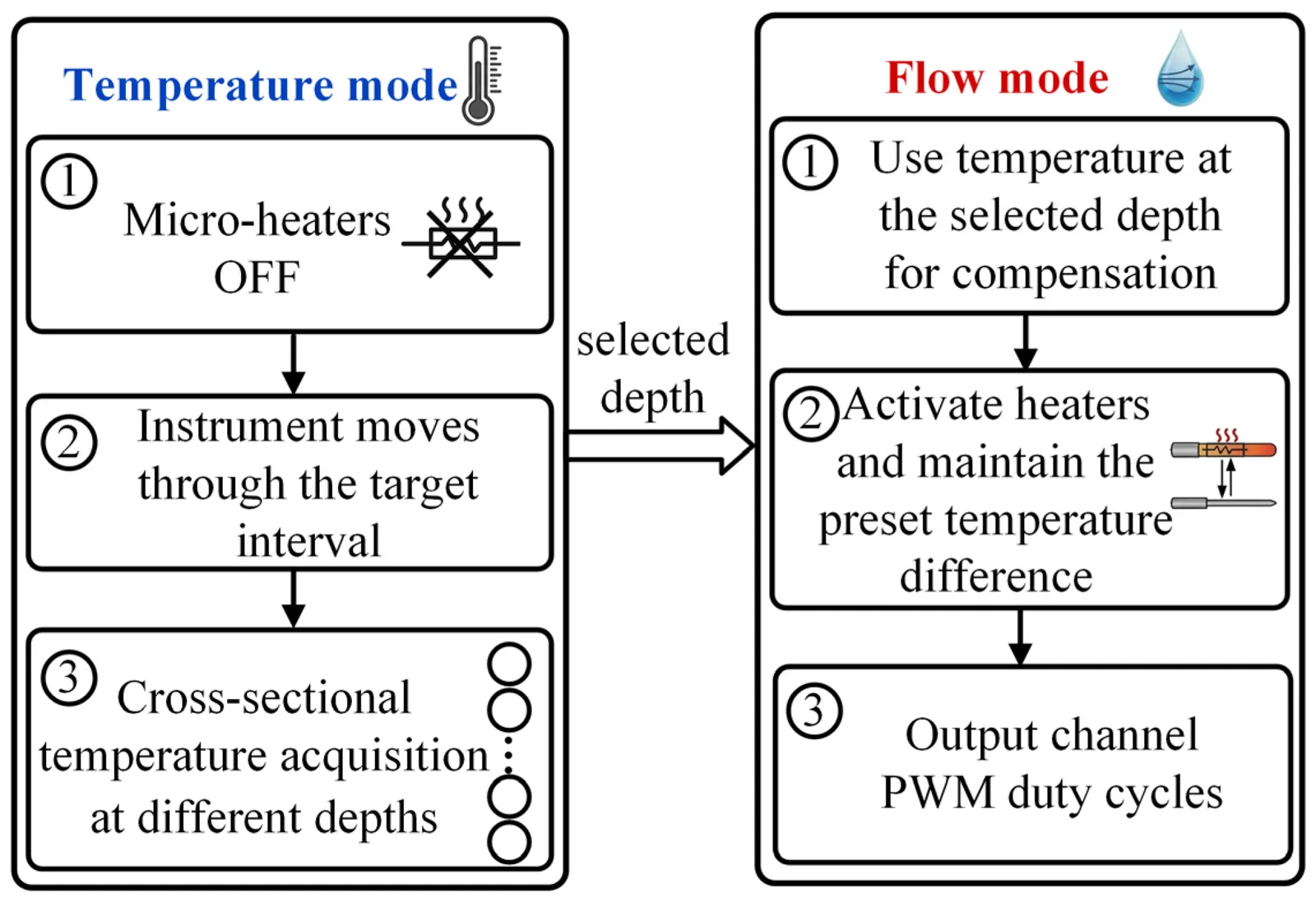
Schematic flowchart of the dual-mode temperature and flow measurement process.

**Figure 3:**
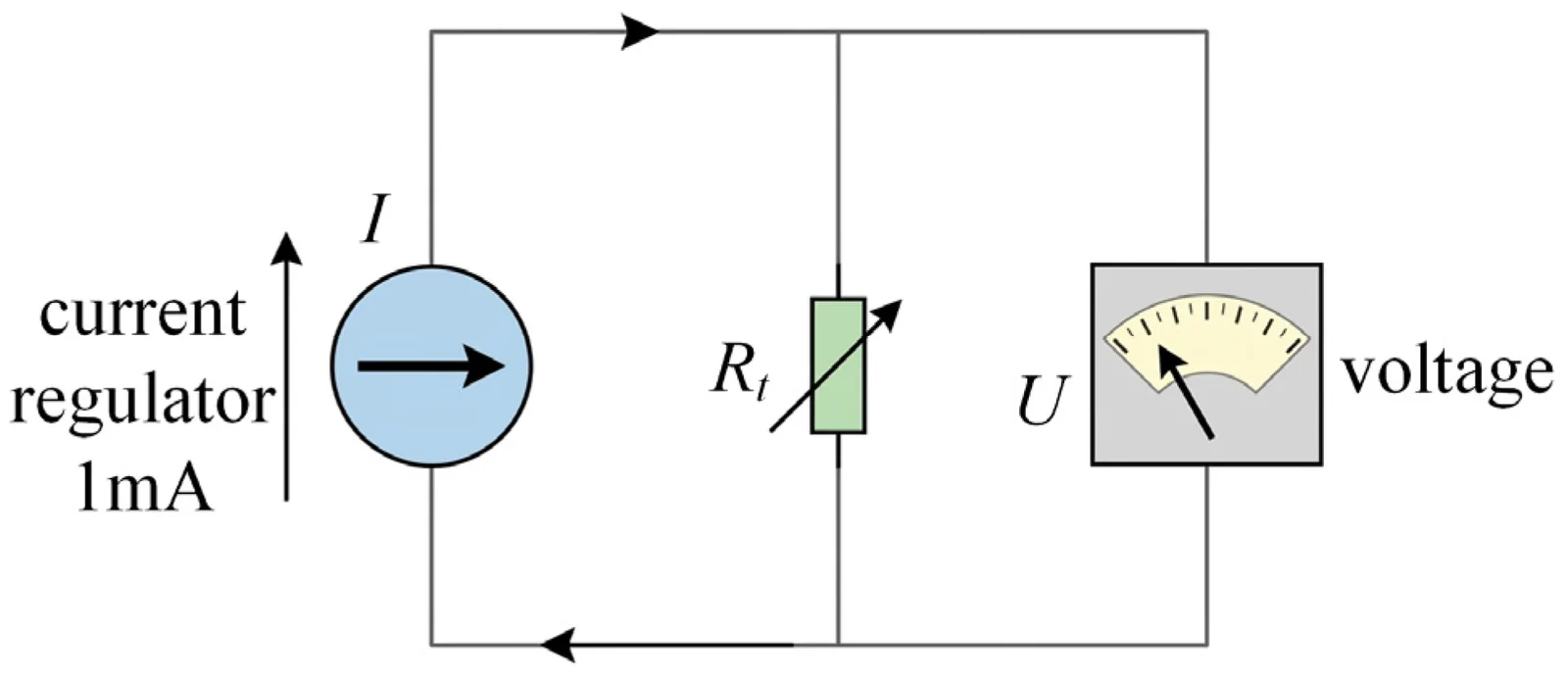
Constant-current PT1000 resistance-measurement circuit.

**Figure 4:**
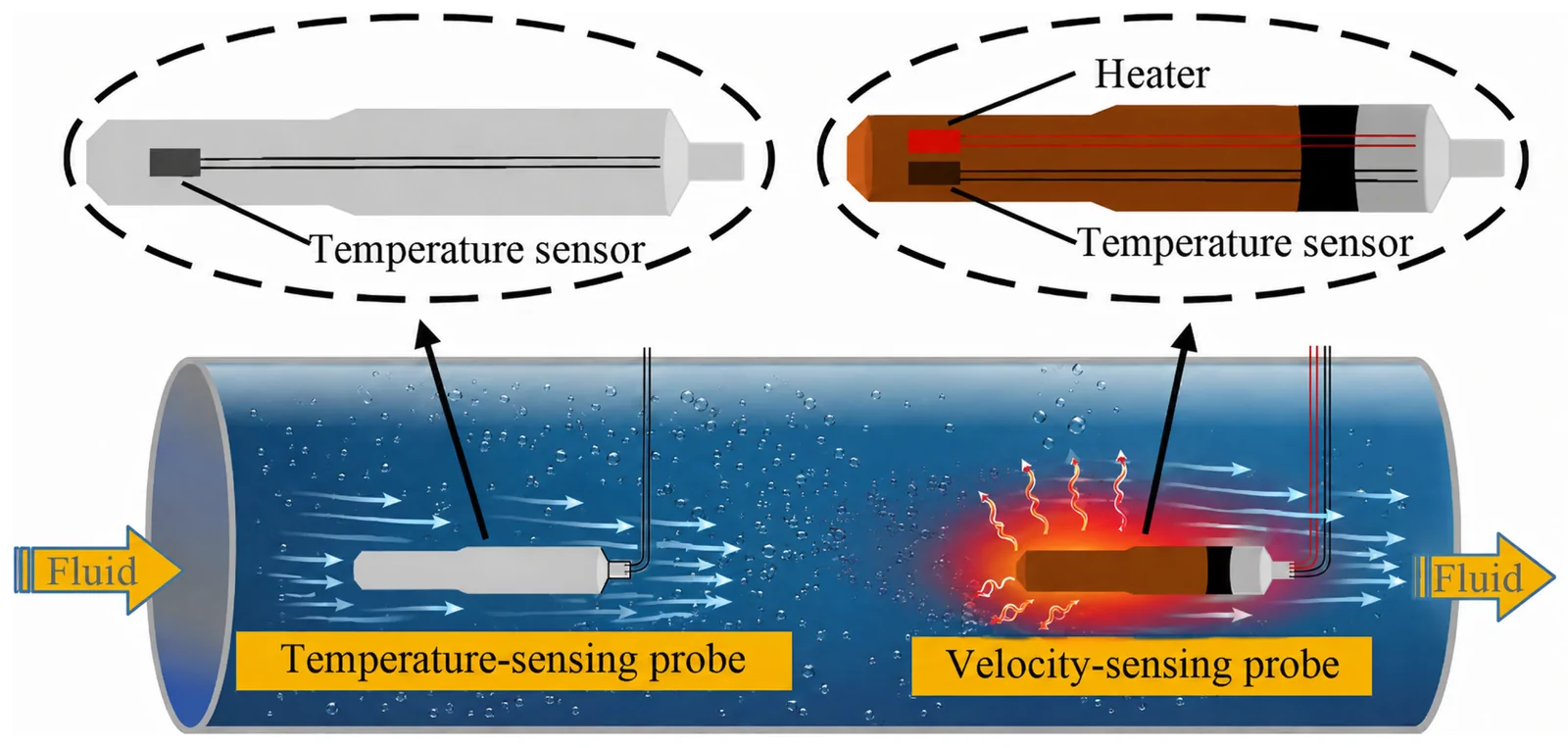
Schematic of the CTD thermal flow-sensing principle.

**Figure 5:**
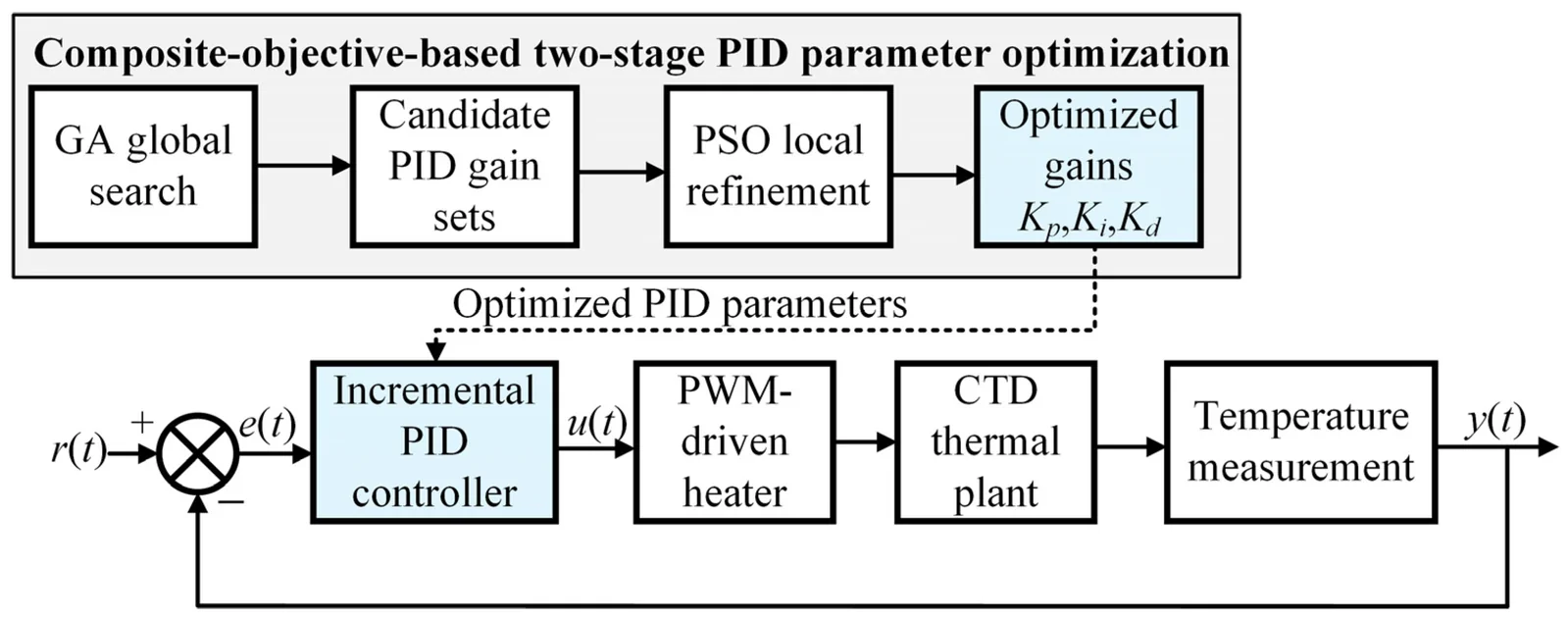
Architecture of the composite-objective-based PSO–GA–PID temperature-control framework.

**Figure 6:**
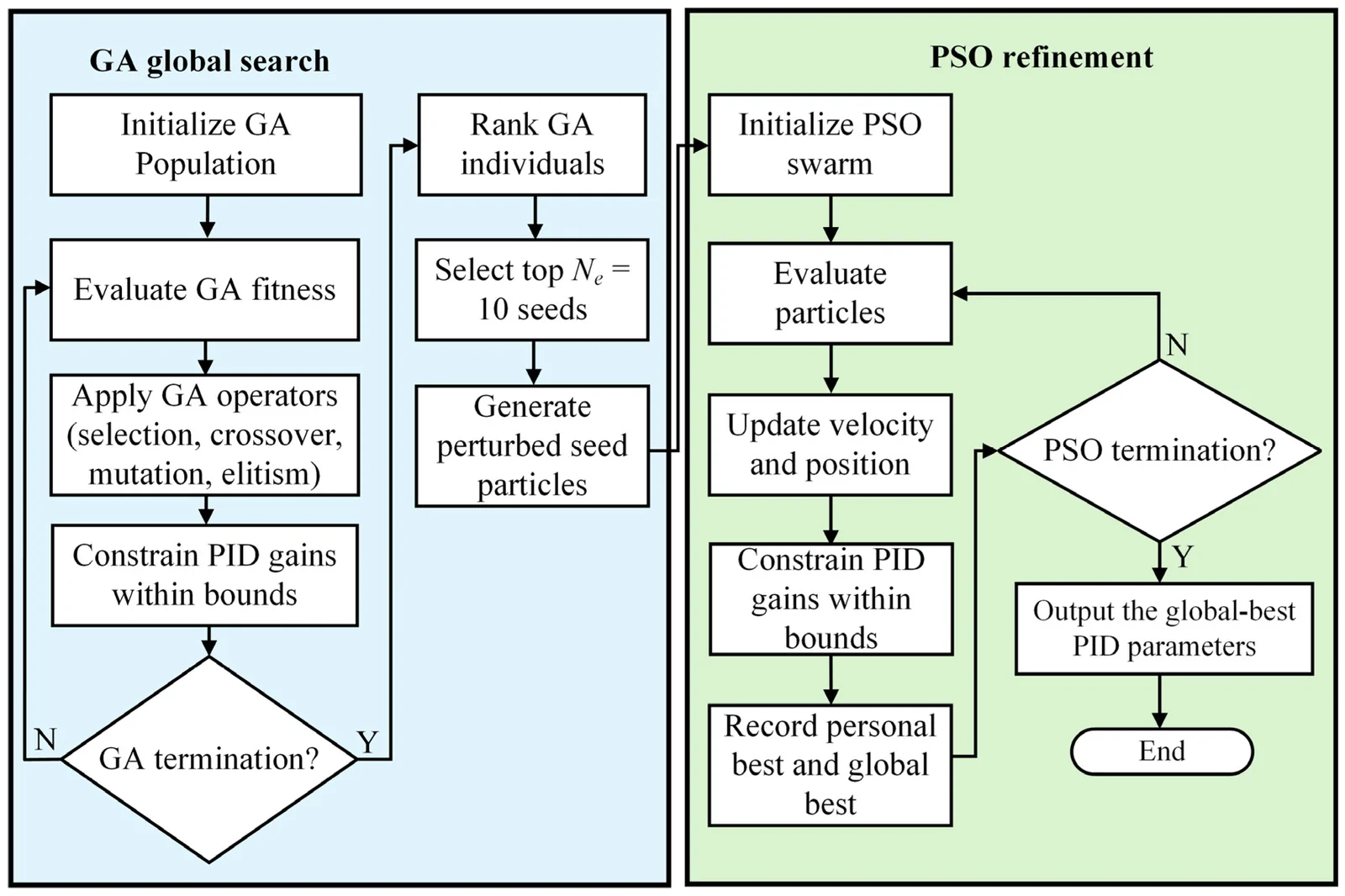
Flowchart of the PSO-enhanced GA optimization procedure.

**Figure 7:**
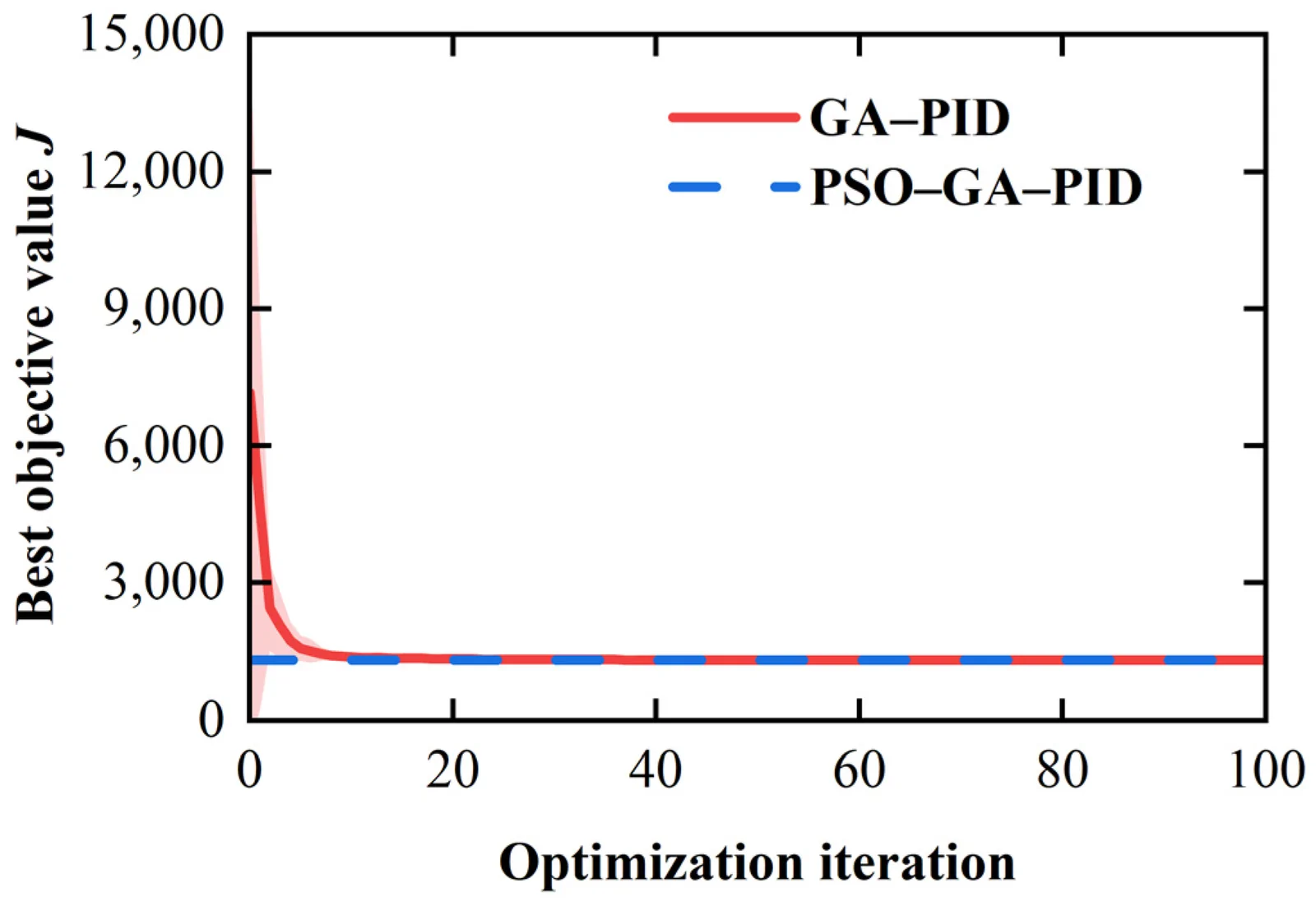
Convergence curves of GA–PID and PSO–GA–PID over 20 independent optimization runs. The shaded bands indicate ±1 standard deviation across the runs.

**Figure 8:**
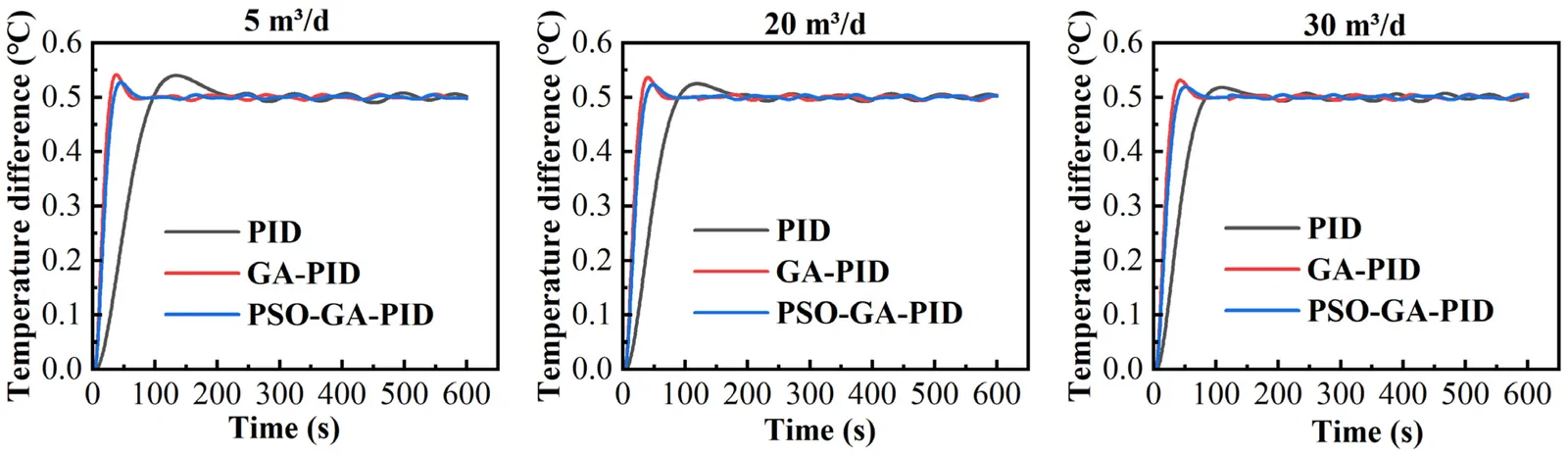
Simulated temperature-difference step responses based on the representative equivalent CTD thermal model at 5, 20, and 30 m^3^/d.

**Figure 9:**
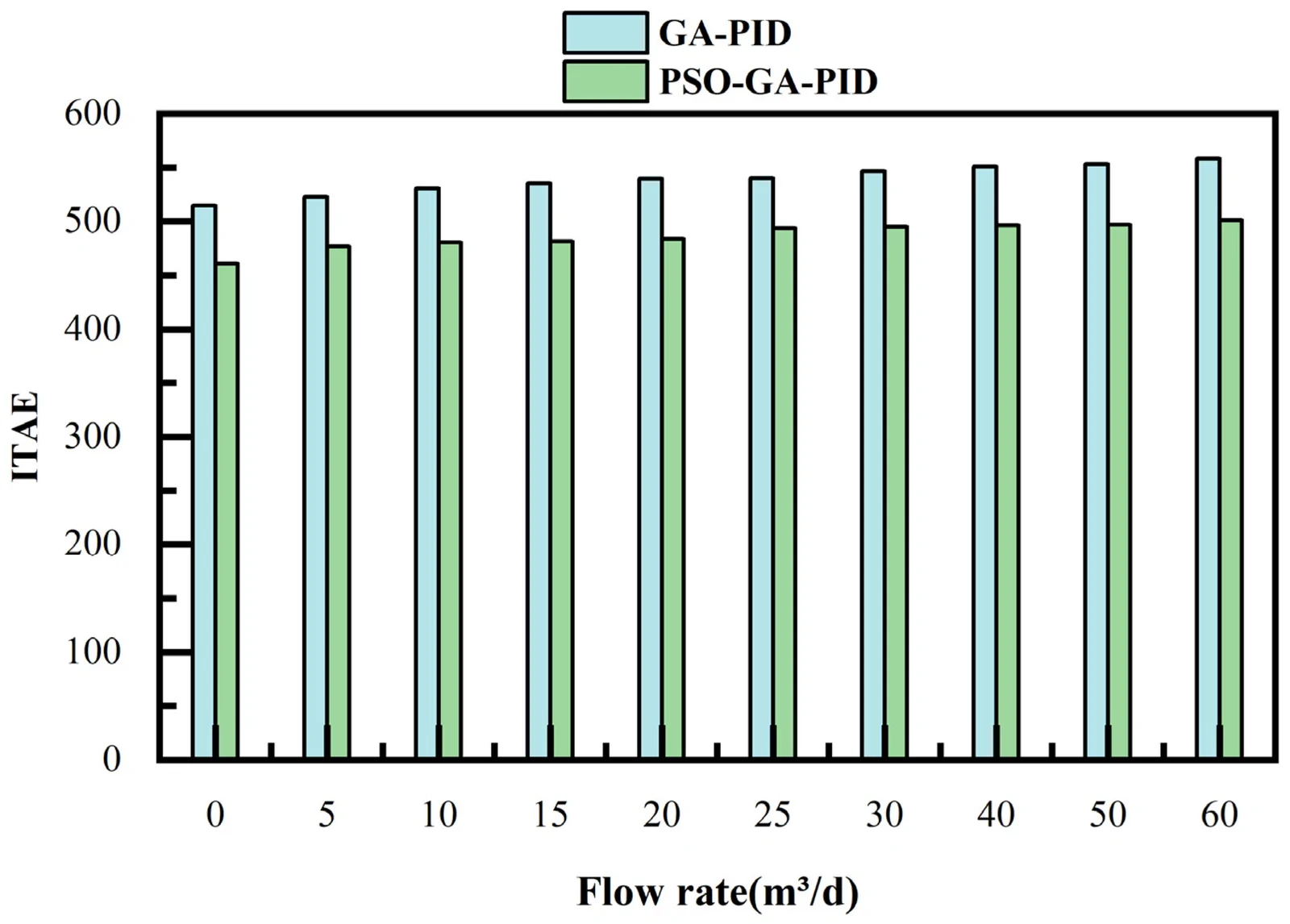
ITAE comparison under the representative equivalent CTD thermal-model conditions.

**Figure 10:**
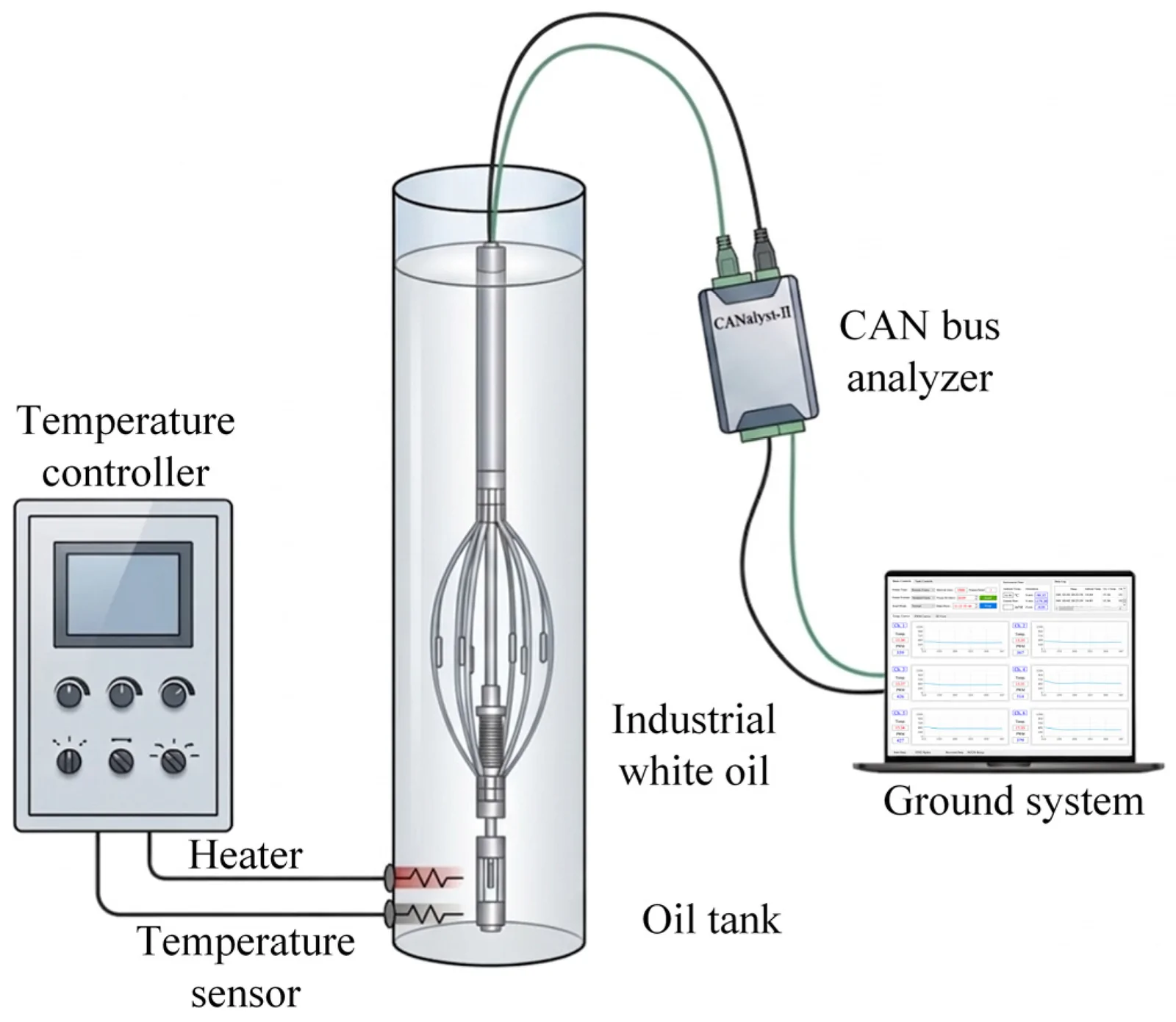
Temperature calibration apparatus.

**Figure 11:**
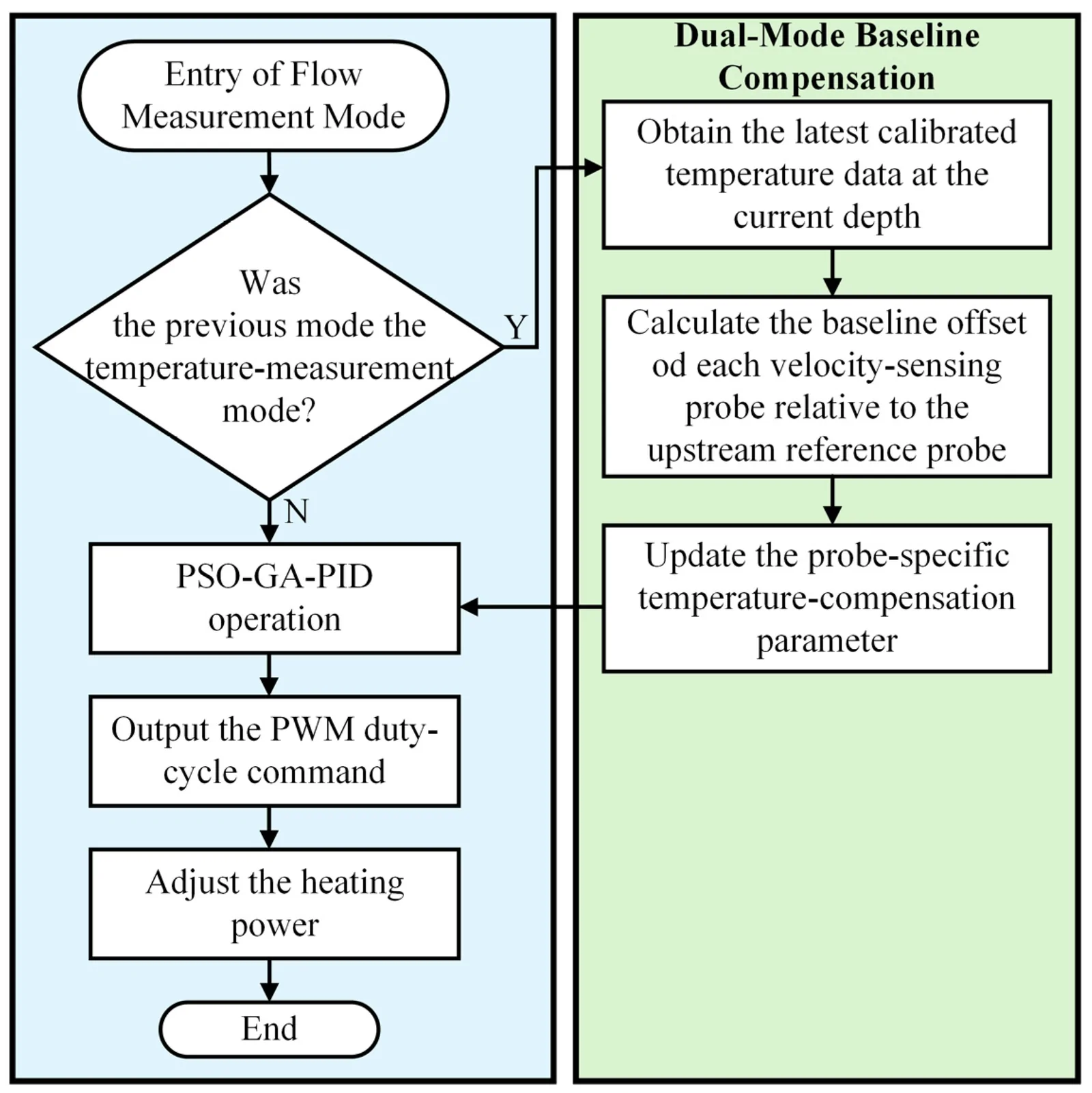
Flowchart of the dual-mode baseline compensation procedure.

**Figure 12:**
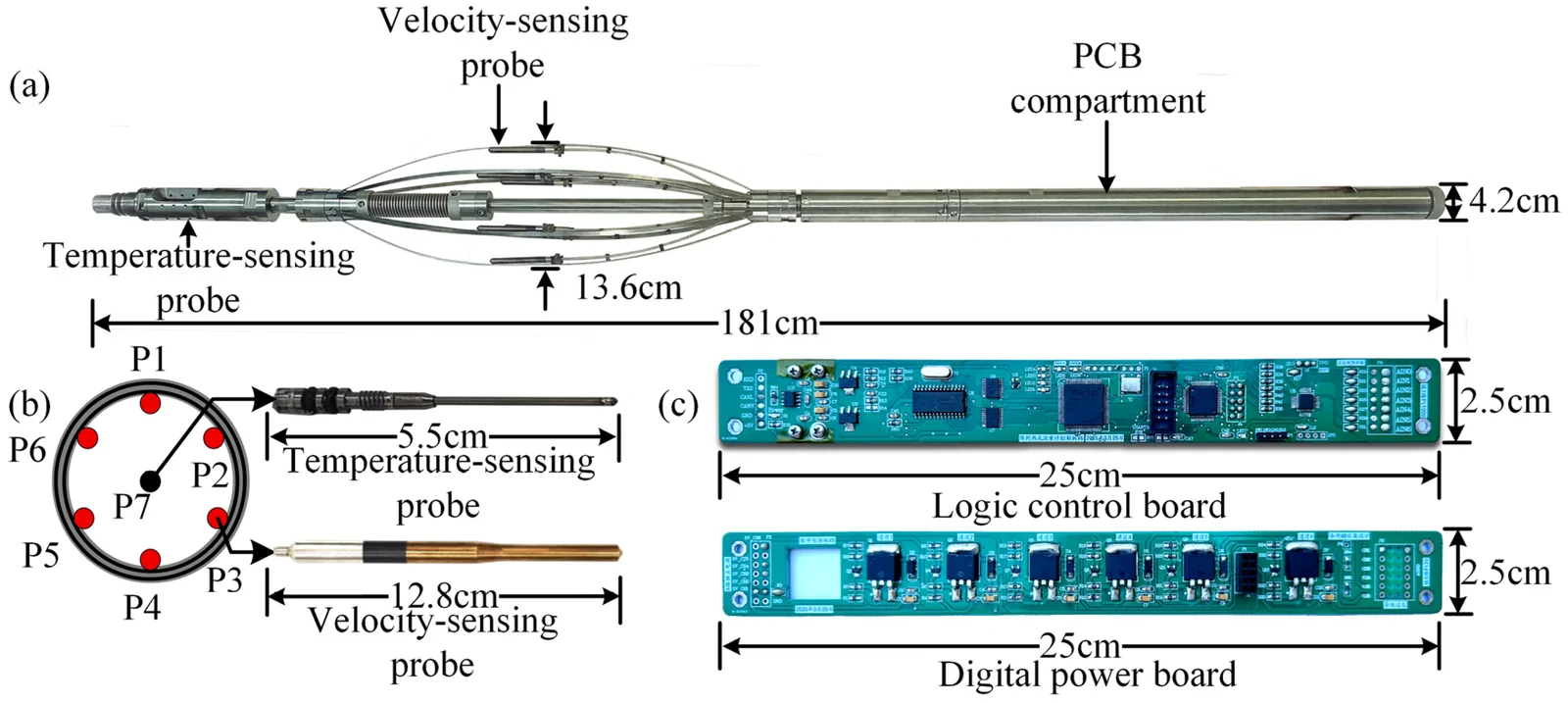
Prototype of the array-based dual-mode temperature–flow logging instrument: (**a**) assembled instrument and dimensions; (**b**) arrangement of the sensing probes and photographs of the temperature- and velocity-sensing probes; (**c**) logic control board and digital power board.

**Figure 13:**
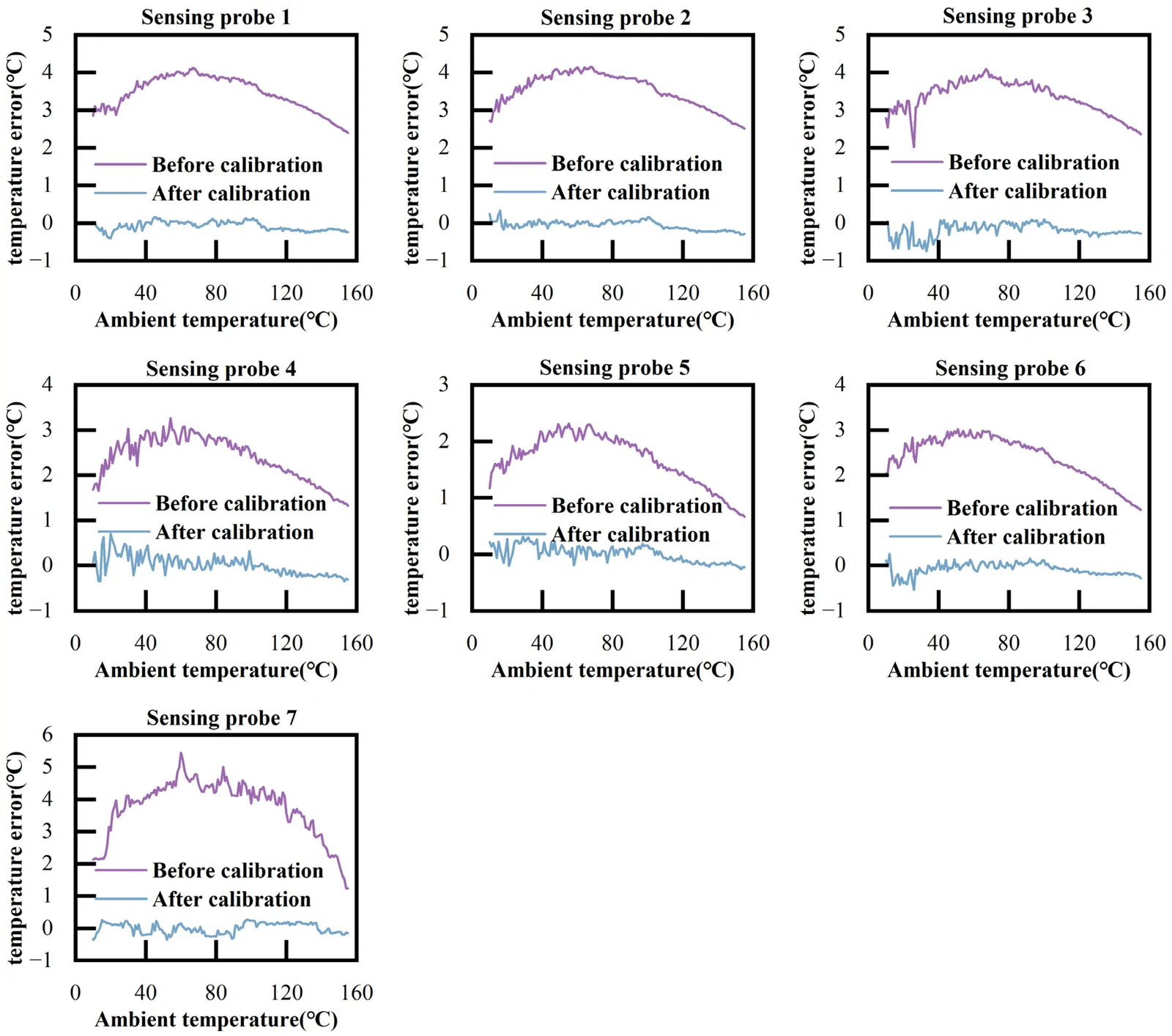
Temperature-error comparison before and after calibration for the seven sensing probes.

**Figure 14:**
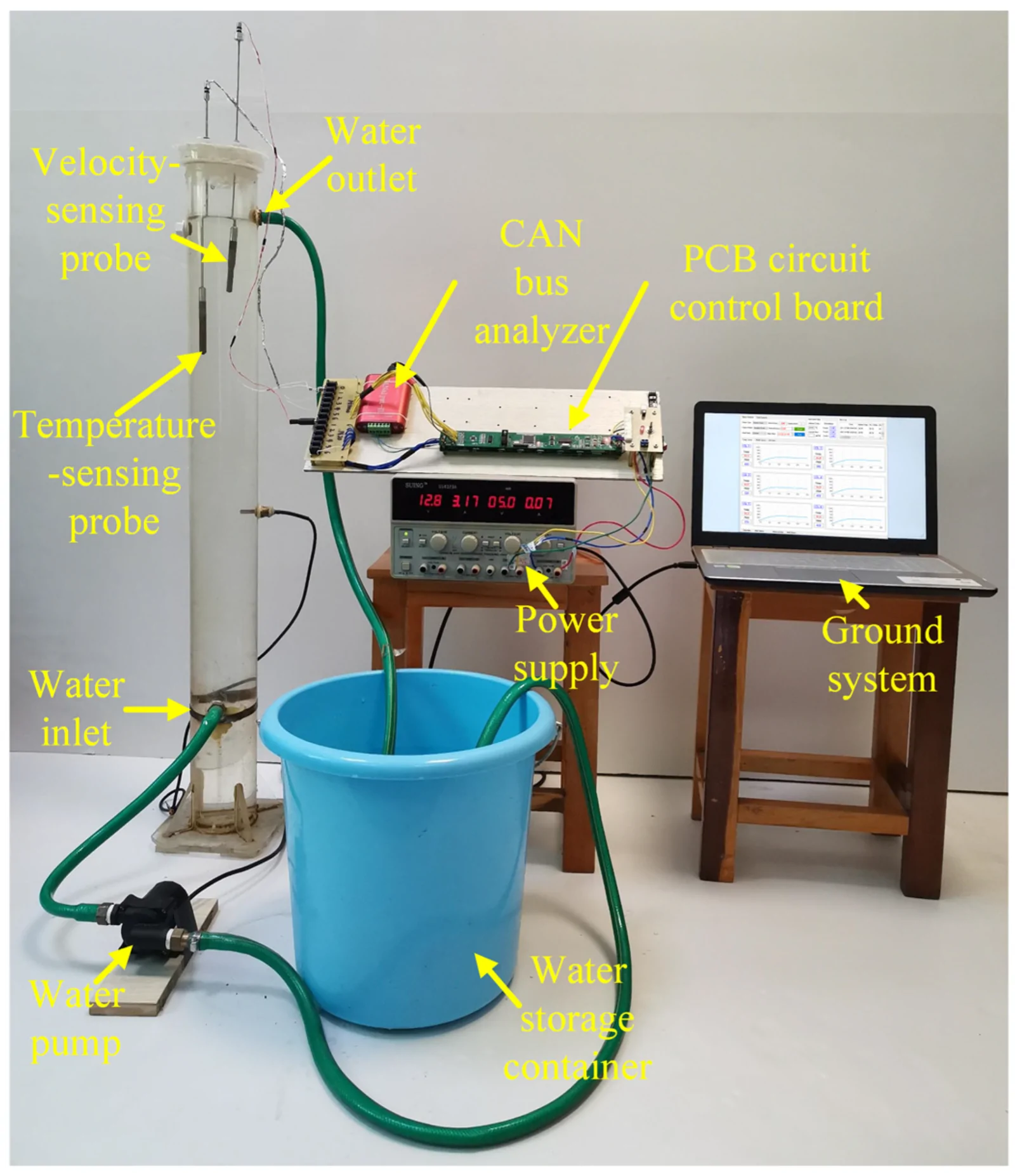
Single-channel CTD temperature-control experimental platform.

**Figure 15:**
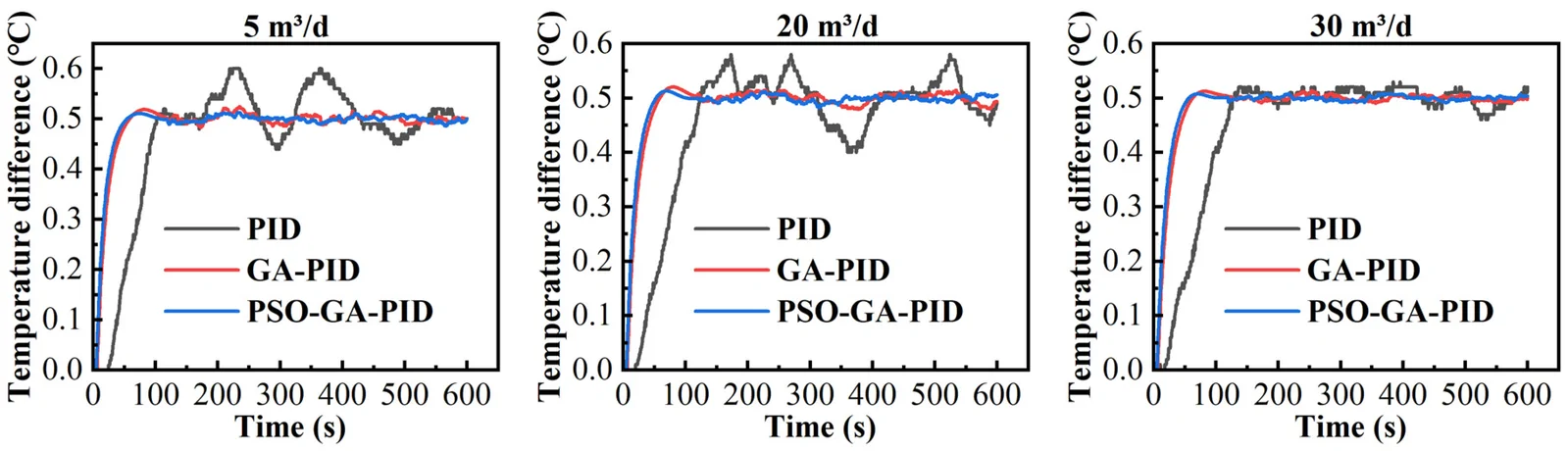
Representative measured temperature-difference step responses at 5, 20, and 30 m^3^/d.

**Figure 16:**
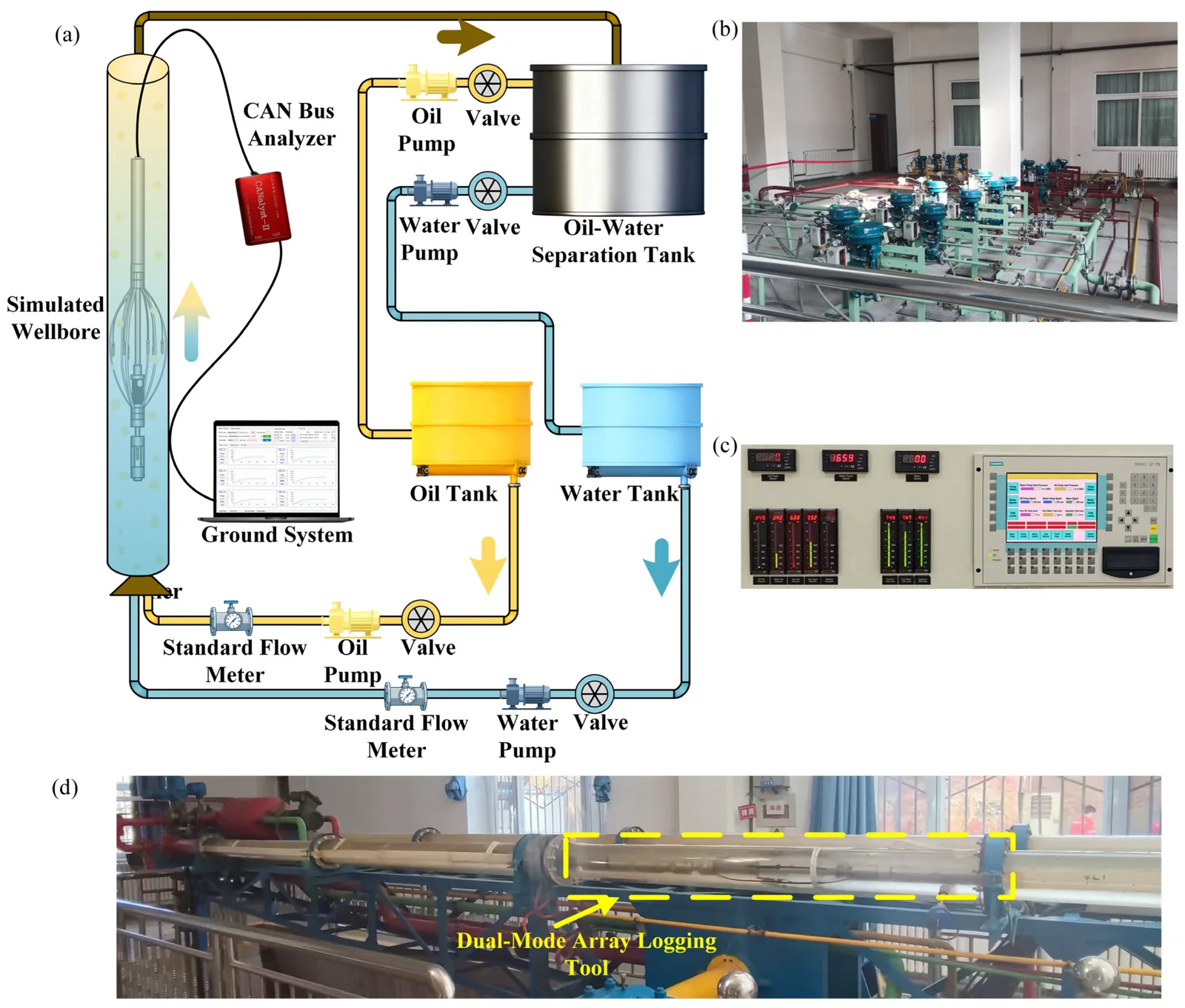
Two-phase flow experimental platform: (**a**) schematic of the CNPC three-phase flow setup; (**b**) flow circulation system; (**c**) control console; (**d**) simulated wellbore with the array-based dual-mode logging instrument.

**Figure 17:**
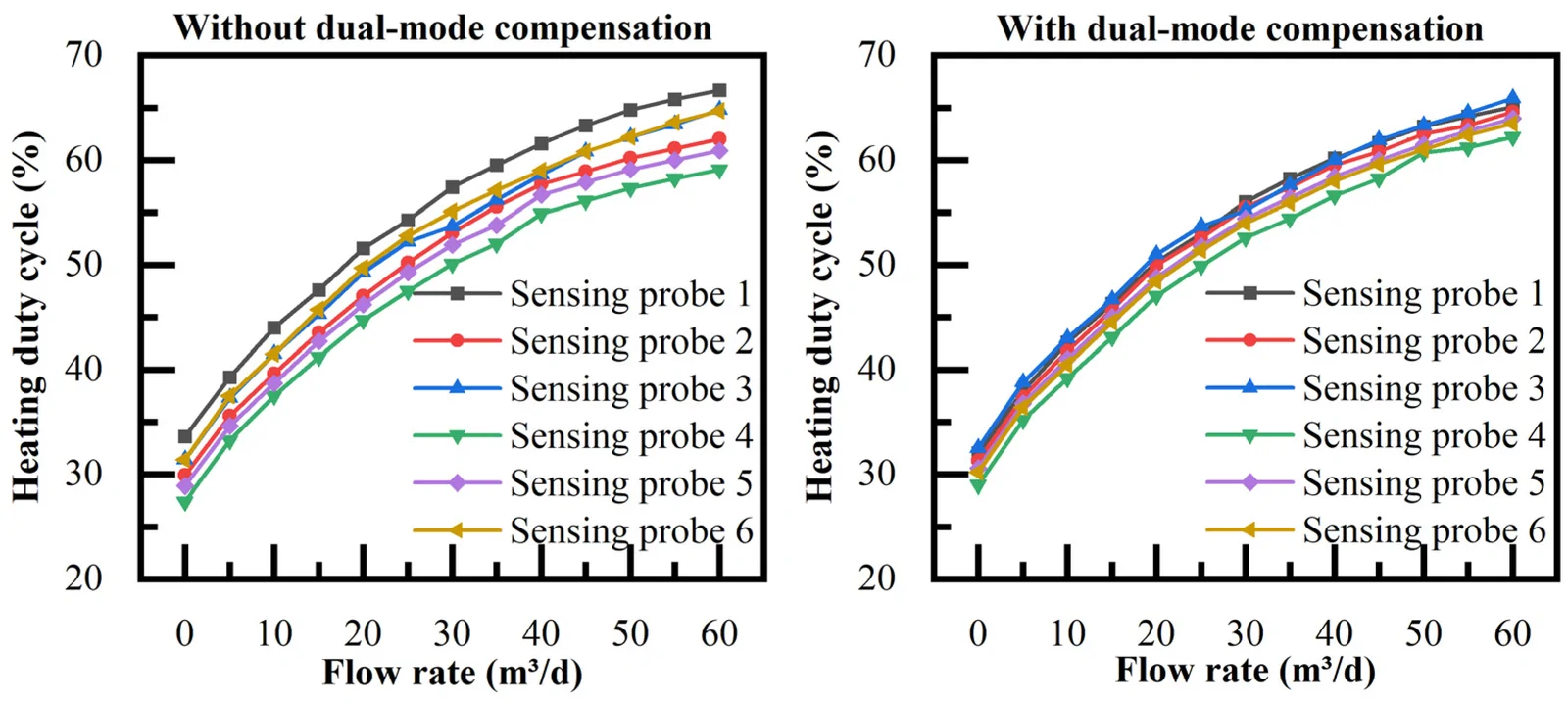
Comparison of heating duty cycles among the six sensing probes under different flow rates: polynomial calibration vs. dual-mode baseline compensation.

**Figure 18:**
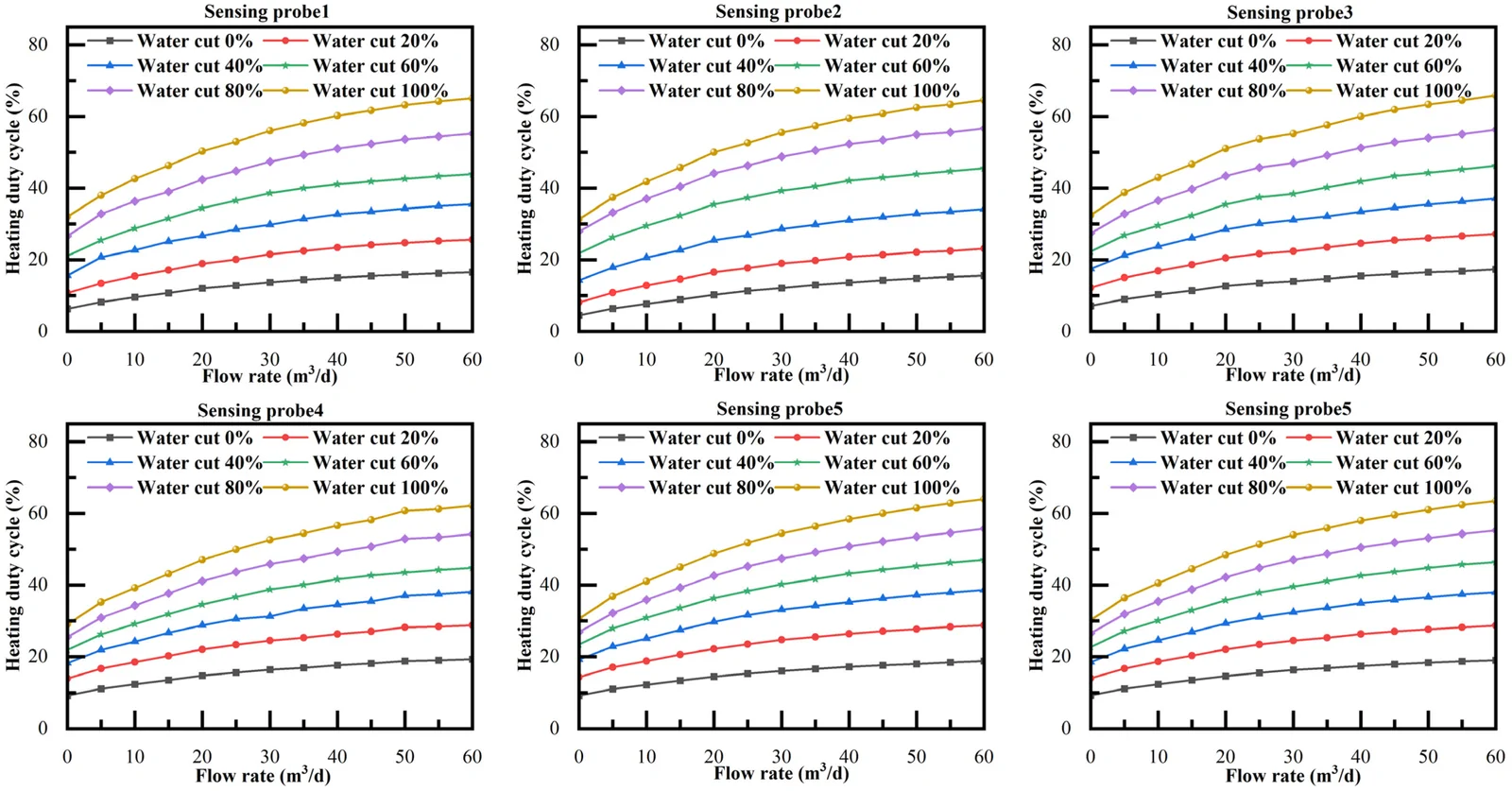
Heating duty-cycle responses of the six sensing probes under different flow rates and water-cut conditions.

**Table 1:** Equivalent thermal-model parameters used in simulation.

Flow Rate (m^3^/d)	*K_q_* (°C/%)	*ω_n_*_,*q*_ (rad/s)	*ζ_q_*	*L_q_* (s)
0	0.0120	0.103	0.26	4.0
5	0.0115	0.110	0.28	4.0
10	0.0109	0.118	0.30	4.0
15	0.0104	0.126	0.32	4.0
20	0.0100	0.135	0.34	4.0
25	0.0096	0.144	0.36	4.0
30	0.0093	0.153	0.38	4.0
40	0.0088	0.168	0.41	4.0
50	0.0084	0.182	0.44	4.0
60	0.0081	0.195	0.47	4.0

**Table 2:** Statistical results of repeated GA–PID and PSO–GA–PID optimization runs.

Method	*K_p_*	*K_i_*	*K_d_*	Final *J*
GA-PID	2.7567 ± 0.2957	1.9495 ± 0.0036	13.3481 ± 1.7198	1329.2275 ± 0.1184
PSO-GA-PID	2.9795 ± 0.0399	1.9525 ± 0.0003	13.4726 ± 0.6473	1329.10748 ± 0.00167

**Table 3:** Quantitative simulation performance indices of the three controllers.

Controller	*K_p_*	*K_i_*	*K_d_*	Mean Rise Time (s)	Mean Settling Time (s)	Mean Overshoot (%)	Maximum Overshoot (%)	Mean ITAE
PID	3.0000	4.0000	0	81.22	108.75	5.300	8.520	1460.37
GA-PID	2.5650	1.9478	12.4813	28.78	49.44	3.993	4.826	539.15
PSO-GA-PID	2.9926	1.9531	12.8764	33.67	39.67	2.341	2.894	486.63

**Table 4:** Fitting equations for each sensing probe.

Probe	Fitting Equation
Velocity-sensing probe 1	*T*_1_ = −5.9 × 10^−7^x^3^ + 6.6364 × 10^−4^x^2^ + 0.9249x − 1.2639
Velocity-sensing probe 2	*T*_2_ = −11.5 × 10^−7^x^3^ + 5.4717 × 10^−4^x^2^ + 0.9398x − 2.0359
Velocity-sensing probe 3	*T*_3_ = −5.8 × 10^−7^x^3^ + 6.6063 × 10^−4^x^2^ + 0.9380x − 1.2459
Velocity-sensing probe 4	*T*_4_ = −17.8 × 10^−7^x^3^ + 6.6914 × 10^−4^x^2^ + 0.9377x − 1.2183
Velocity-sensing probe 5	*T*_5_ = −5.9 × 10^−7^x^3^ + 6.6066 × 10^−4^x^2^ + 0.9080x − 1.2458
Velocity-sensing probe 6	*T*_6_ = −5.4 × 10^−7^x^3^ + 6.6462 × 10^−4^x^2^ + 0.7686x − 1.2538
Temperature-sensing probe	*T*_7_ = −5.2 × 10^−7^x^3^ + 6.4163 × 10^−4^x^2^ + 0.9096x − 1.2875

**Table 5:** Fitting and calibration metrics for each sensing probe.

Probe	*R* ^2^	RMSE (°C)
Velocity-sensing probe 1	0.999959	0.269274
Velocity-sensing probe 2	0.999996	0.081269
Velocity-sensing probe 3	0.999958	0.268663
Velocity-sensing probe 4	0.999991	0.122174
Velocity-sensing probe 5	0.999991	0.122172
Velocity-sensing probe 6	0.999991	0.122171
Temperature-sensing probe	0.999958	0.269721

**Table 6:** Repeatability of calibrated temperature measurements from three independent temperature cycles.

Ambient Temperature (°C)	Mean SD Across Probes (°C)	Maximum SD (°C)	Maximum Range (°C)
20	0.043	0.061	0.120
60	0.036	0.052	0.100
100	0.029	0.043	0.080
150	0.032	0.046	0.090

**Table 7:** Quantitative experimental performance indices of the three controllers.

Controller	*K_p_*	*K_i_*	*K_d_*	Mean Rise Time (s)	Mean Settling Time (s)	Mean Overshoot (%)	Maximum Overshoot (%)	Mean ITAE
PID	3.0000	4.0000	0	112.67	552.43	14.00	20.00	5590.05
GA-PID	2.5650	1.9478	12.4813	49.72	68.19	7.95	8.96	1231.84
PSO-GA-PID	2.9926	1.9531	12.8764	42.98	56.41	4.34	5.66	807.08

**Table 8:** Repeatability of measured CTD control performance at representative flow-rate conditions (mean ± sample SD across three independent heating runs).

Flow Rate (m^3^/d)	Controller	*n*	Rise Time (s)	Settling Time (s)	Overshoot (%)	ITAE
5	GA–PID	3	52.33 ± 0.58	70.67 ± 1.53	8.93 ± 0.34	1369.03 ± 8.26
5	PSO–GA–PID	3	45.33 ± 2.31	57.67 ± 0.58	5.24 ± 0.10	875.52 ± 24.62
20	GA–PID	3	48.67 ± 2.08	68.67 ± 0.58	7.97 ± 0.23	1242.41 ± 14.91
20	PSO–GA–PID	3	41.00 ± 3.46	58.67 ± 1.15	4.24 ± 0.15	815.24 ± 22.05
30	GA–PID	3	48.67 ± 0.58	64.67 ± 0.58	7.68 ± 0.39	1161.98 ± 19.93
30	PSO–GA–PID	3	43.00 ± 2.00	53.67 ± 1.15	4.12 ± 0.40	762.02 ± 13.95

**Table 9:** Heating duty-cycle responses of the six sensing probes before and after dual-mode baseline compensation.

Flow Rate (m^3^/d)	Heating Duty Cycle (*%*)							
	Probe 1	Probe 2	Probe 3	Probe 4	Probe 5	Probe 6	Mean	Maximum Difference
0	33.6/32.0	29.9/31.3	31.4/32.5	27.4/29.0	28.9/30.6	31.4/30.2	30.4/30.9	6.2/3.5
5	39.3/38.0	35.6/37.4	37.3/38.8	33.2/35.2	34.6/36.8	37.5/36.4	36.3/37.1	6.1/3.6
10	44.0/42.6	39.6/41.8	41.5/43.0	37.5/39.2	38.7/41.0	41.5/40.5	40.5/41.3	6.5/3.8
15	47.6/46.3	43.5/45.7	45.3/46.7	41.2/43.1	42.7/45.0	45.7/44.5	44.3/45.2	6.4/3.6
20	51.6/50.3	47.0/50.0	49.3/51.0	44.7/47.0	46.2/48.8	49.7/48.4	48.1/49.2	6.9/4.0
25	54.3/53.0	50.2/52.6	52.2/53.7	47.5/49.9	49.3/51.8	52.8/51.4	51.1/52.1	6.8/3.8
30	57.4/55.8	53.1/55.5	53.7/55.2	50.1/52.6	51.9/54.4	55.1/54.0	53.5/54.6	7.3/3.2
35	59.5/58.2	55.6/57.4	56.2/57.6	52.0/54.4	53.8/56.4	57.1/55.9	55.7/56.6	7.5/3.8
40	61.6/60.2	57.7/59.5	58.6/60.0	54.9/56.6	56.7/58.4	59.0/58.0	58.1/58.8	6.7/3.6
45	63.3/61.7	58.9/60.8	60.8/61.9	56.1/58.2	57.9/60.0	60.8/59.6	59.6/60.4	7.2/3.7
50	64.8/63.2	60.2/62.5	62.2/63.3	57.3/60.7	59.1/61.5	62.2/61.0	60.9/62.0	7.5/2.6
55	65.8/64.2	61.1/63.3	63.4/64.5	58.2/61.2	60.0/62.8	63.6/62.4	62.0/63.1	7.6/3.3
60	66.7/65.1	62.0/64.6	64.8/65.9	59.1/62.2	60.9/64.0	64.7/63.5	63.0/64.2	7.6/3.7

Notes: Data in red and blue indicate heating duty cycles obtained using polynomial calibration only and dual-mode baseline compensation, respectively.

**Table 10:** Repeatability of steady-state array duty-cycle responses with and without dual-mode baseline compensation (mean ± sample SD across three independent measurements).

Flow Rate (m^3^/d)	Compensation Condition	*n*	Array Mean Duty Cycle (%)	Maximum Inter-Probe Difference (Percentage Points)
0	Polynomial calibration only	3	30.45 ± 0.10	6.17 ± 0.40
0	Polynomial calibration + baseline compensation	3	30.95 ± 0.10	3.47 ± 0.31
20	Polynomial calibration only	3	48.10 ± 0.10	6.87 ± 0.40
20	Polynomial calibration + baseline compensation	3	49.27 ± 0.10	3.97 ± 0.31
30	Polynomial calibration only	3	53.57 ± 0.10	7.27 ± 0.40
30	Polynomial calibration + baseline compensation	3	54.60 ± 0.10	3.17 ± 0.40

**Table 11:** Principal quantified error sources and associated standard uncertainties in the CTD measurement chain.

Source	Specification or Measured Value	Standard Uncertainty	Evaluation Basis
Reference temperature	±0.10 °C	0.0577 °C	0.10√3
Calibrated temperature response	±0.60 °C at 10–60 °C; ±0.30 °C at 60–150 °C	0.346 °C; 0.173 °C	Conservative estimate based on maximum observed residual
IDAC current stability	≤0.10% of nominal current	0.0577%	0.10%/√3
ADC quantization	24 bit	*q*_ADC_/√12	*q*_ADC_ = *V*_FS_/2^24^
PWM duty-cycle quantization	0.1 percentage points	0.0289 percentage points	0.10/√12
Heater resistance variation	22.207 ± 0.466 Ω	0.466 Ω	Sample SD of six measured heater resistances
Reference flow rate	±0.5% of reading	0.289% of reading	0.5%/√3

Notes: The heater resistance value represents the measured inter-probe variation rather than the uncertainty of resistance measurement.

## Data Availability

The data presented in this study are available on request from the corresponding author.

## Nomenclature

Symbols and Variables		
**Temperature and Heat Transfer**		
**Symbol**	**Description**	**Unit**
*T*; *T_j_*	Temperature; temperature indicated by sensing probe j	°C
*T_m_*_,*i*_; *T_n_*	Temperature of heated probe i; upstream reference-probe temperature	°C
*T_i_*(*z*); *T_7_*(*z*)	Calibrated temperature of probe i and reference probe P7 at depth z	°C
Δ*T_i_*; Δ*T_set_*	Heated-to-reference temperature difference; prescribed CTD setpoint	°C
Δ*T_c_*_,*i*_; Δ*T_e_*	Preheating probe-to-reference baseline offset; unheated temperature-difference bias	°C
*z*; *t*	Measurement depth; elapsed time	m; s
*R_T_*; *R*_0_	PT1000 resistance at temperature T; nominal resistance at 0 °C	Ω
*A*; *B*; *C*	Callendar–Van Dusen coefficients	°C^−1^; °C^−2^; °C^−4^
*I_s_*; *U_s_*_,*j*_	PT1000 excitation current; measured voltage across probe j	A; V
*P_i_*; *R_i_*	Heating power; resistance of heater i	W; Ω
*U_t_*; *U_c_*_,*i*_	Heater on-state voltage; RMS-equivalent heater voltage	V
*θ_i_*	PWM duty cycle of heated probe i; θ_i_/100 is the duty ratio	%
*h_i_*; *S_i_*	Convective heat-transfer coefficient; effective probe heat-transfer area	W·m^−2^·K^−1^; m^2^
*l_i_*; *d_i_*	Effective heated-section length; probe diameter	m
*Nu_i_*; *Re_i_*; *Pr*	Nusselt number; Reynolds number; Prandtl number	–
*λ_f_*; *μ*	Fluid thermal conductivity; dynamic viscosity	W·m^−1^·K^−1^; Pa·s
*ρ*; *c_p_*; *v_i_*	Fluid density; specific heat capacity; local flow velocity	kg·m^−3^; J·kg^−1^·K^−1^; m·s^−1^
*k*_1_*_i_*; *k*_2_*_i_*	Probe-specific effective coefficients in the CTD heat-transfer relationship	W·°C^−1^; W·°C^−1^/(m·s^−1^)^1/2^
*A*_1_*_i_*; *A*_2_*_i_*; *A*_3_*_i_*	Probe-specific duty-cycle-to-velocity calibration coefficients	m·s^−1^·%^−2^; m·s^−1^·%^−1^; m·s^−1^
**Control, Optimization, and Simulation**		
**Symbol**	**Description**	**Unit**
*Q_q_*; *q*	Prescribed volumetric flow rate for operating condition q; condition index	m^3^·d^−1^; –
*r*(*t*); *y_q_*(*t*)	Reference temperature-difference signal; equivalent plant output	°C
*e*(*t*); *e_q_*(*t*)	Temperature-difference tracking error, r(t) − yq(t)	°C
*u*(*t*); *u_q_*(*t*)	Actuator input expressed as the PWM duty-cycle command	%
*G_q_*(*s*); *s*	Equivalent CTD plant transfer function at condition q; Laplace variable	°C/%; s^−1^
*K_q_*; *ω_n_*_,*q*_	Equivalent plant gain; natural angular frequency	°C/%; rad·s^−1^
*ζ_q_*; *L_q_*	Equivalent damping ratio; pure time delay	–; s
*K_p_*; *K_i_*; *K_d_*	Proportional, integral, and derivative PID gains	%·°C^−1^; %·°C^−1^·s^−1^; %·s·°C^−1^
*T_s_*; *t_k_*; *N*	Controller sampling period; time at sample k; number of samples	s; s; –
*k*; *d*	Discrete-time sample index; delay in samples	–
*x*_1_; *x*_2_	Continuous plant states yq and the time derivative of yq	°C; °C·s^−1^
*J*(*x*); *x*	Composite optimization objective; PID parameter vector	–; mixed
*ITAE*; *M_p_*; *M_p_*_,*max*_	Integral of time-weighted absolute error; overshoot; maximum overshoot	°C·s^2^; %; %
*D_u_*	PWM-output variation term	%^2^
*w_os_*_,*m*_; *w_os_*_,*max*_; *w_du_*	Weighting coefficients in the composite objective	Term-dependent
*x_i_^ξ^*; *v_i_^ξ^*	Position and velocity of particle i at optimization iteration ξ	Search-space units
*p_i_*; *g*	Personal-best position; global-best position of PSO	Search-space units
*c*_1_; *c*_2_; *r*_1_; *r*_2_	PSO acceleration coefficients; independent uniform random variables	–
*w_ξ_*; *w_max_*; *w_min_*	PSO inertia weight; initial and final inertia weights	–
*ξ*; *K_max_*	Optimization iteration index; maximum number of PSO iterations	–
**Calibration, Repeatability, and Uncertainty**		
**Symbol**	**Description**	**Unit**
*x*; *α*_0_–*α*_3_	Raw probe temperature used in cubic regression; polynomial coefficients	°C; coefficient-specific
*R*^2^; *RMSE*	Coefficient of determination; root-mean-square calibration error	–; °C
*n*; *SD*	Number of independent runs; sample standard deviation	–; same as measured variable
*a*; Δ*x*	Half-width of a symmetric tolerance; digital quantization step	Units of the corresponding quantity
*u*(*x*); *u_q_*(*x*)	Standard uncertainty of x; quantization standard uncertainty	Units of x
*u_R_*; *U*; *I*; *R*	Resistance standard uncertainty; measured voltage, current, and resistance	Ω; V; A; Ω
*q_ADC_*; *V_FS_*	ADC quantization step; effective ADC full-scale input voltage	V
$\bar{R}$; *_sR_*	Mean heater resistance; sample standard deviation of heater resistance	Ω
Δ*t*	Actual acquisition interval used for experimental ITAE	s
**Abbreviation**	**Full Term**	
ADC	Analog-to-digital converter	
BN	Boron nitride	
CCS	Constant-current source	
CTD	Constant temperature difference	
DSP	Digital signal processor	
GA	Genetic algorithm	
ITAE	Integral of time-weighted absolute error	
MEMS	Microelectromechanical systems	
PID	Proportional–integral–derivative	
PSO	Particle swarm optimization	
PT1000	Platinum resistance thermometer with a nominal resistance of 1000 Ω at 0 °C	
PWM	Pulse-width modulation	
RMS	Root mean square	
RMSE	Root mean square error	
SD	Standard deviation	
